# Cardiac-specific PFKFB3 overexpression prevents diabetic cardiomyopathy via enhancing OPA1 stabilization mediated by K6-linked ubiquitination

**DOI:** 10.1007/s00018-024-05257-5

**Published:** 2024-05-22

**Authors:** Jinlan Luo, Shuiqing Hu, Jingrui Liu, Lili Shi, Liman Luo, Wenhua Li, Yueting Cai, Jiaxin Tang, Siyang Liu, Menglu Fu, Ruolan Dong, Yan Yang, Ling Tu, Xizhen Xu

**Affiliations:** 1 Department of Geriatric Medicine, Tongji Hospital, Tongji Medical College, Huazhong University of Science and Technology, Wuhan, 430030 China; 2 Division of Cardiology and Department of Internal Medicine, Tongji Hospital, Tongji Medical College, Huazhong University of Science and Technology, Wuhan, 430030 China; 3 Institute of Integrated Traditional Chinese and Western Medicine, Tongji Hospital, Tongji Medical College, Huazhong University of Science and Technology, Wuhan, 430030 China; 4 Health Management Center, Tongji Hospital, Tongji Medical College, Huazhong University of Science and Technology, Wuhan, 430030 China; 5 Hubei Key Laboratory of Genetics and Molecular Mechanisms of Cardiological Disorders, Wuhan, 430030 China

**Keywords:** Heart failure, Mitochondrial dysfunction, Protein–protein interactions, rAAV9, Therapeutics

## Abstract

Diabetic cardiomyopathy (DCM) is a prevalent complication of type 2 diabetes (T2D). 6-phosphofructo-2-kinase/fructose-2,6-biphosphatase 3 (PFKFB3) is a glycolysis regulator. However, the potential effects of PFKFB3 in the DCM remain unclear. In comparison to db/m mice, PFKFB3 levels decreased in the hearts of db/db mice. Cardiac-specific PFKFB3 overexpression inhibited myocardial oxidative stress and cardiomyocyte apoptosis, suppressed mitochondrial fragmentation, and partly restored mitochondrial function in db/db mice. Moreover, PFKFB3 overexpression stimulated glycolysis. Interestingly, based on the inhibition of glycolysis, PFKFB3 overexpression still suppressed oxidative stress and apoptosis of cardiomyocytes in vitro, which indicated that PFKFB3 overexpression could alleviate DCM independent of glycolysis. Using mass spectrometry combined with co-immunoprecipitation, we identified optic atrophy 1 (OPA1) interacting with PFKFB3. In db/db mice, the knockdown of OPA1 receded the effects of PFKFB3 overexpression in alleviating cardiac remodeling and dysfunction. Mechanistically, PFKFB3 stabilized OPA1 expression by promoting E3 ligase NEDD4L-mediated atypical K6-linked polyubiquitination and thus prevented the degradation of OPA1 by the proteasomal pathway. Our study indicates that PFKFB3/OPA1 could be potential therapeutic targets for DCM.

**Graphical Abstract:**

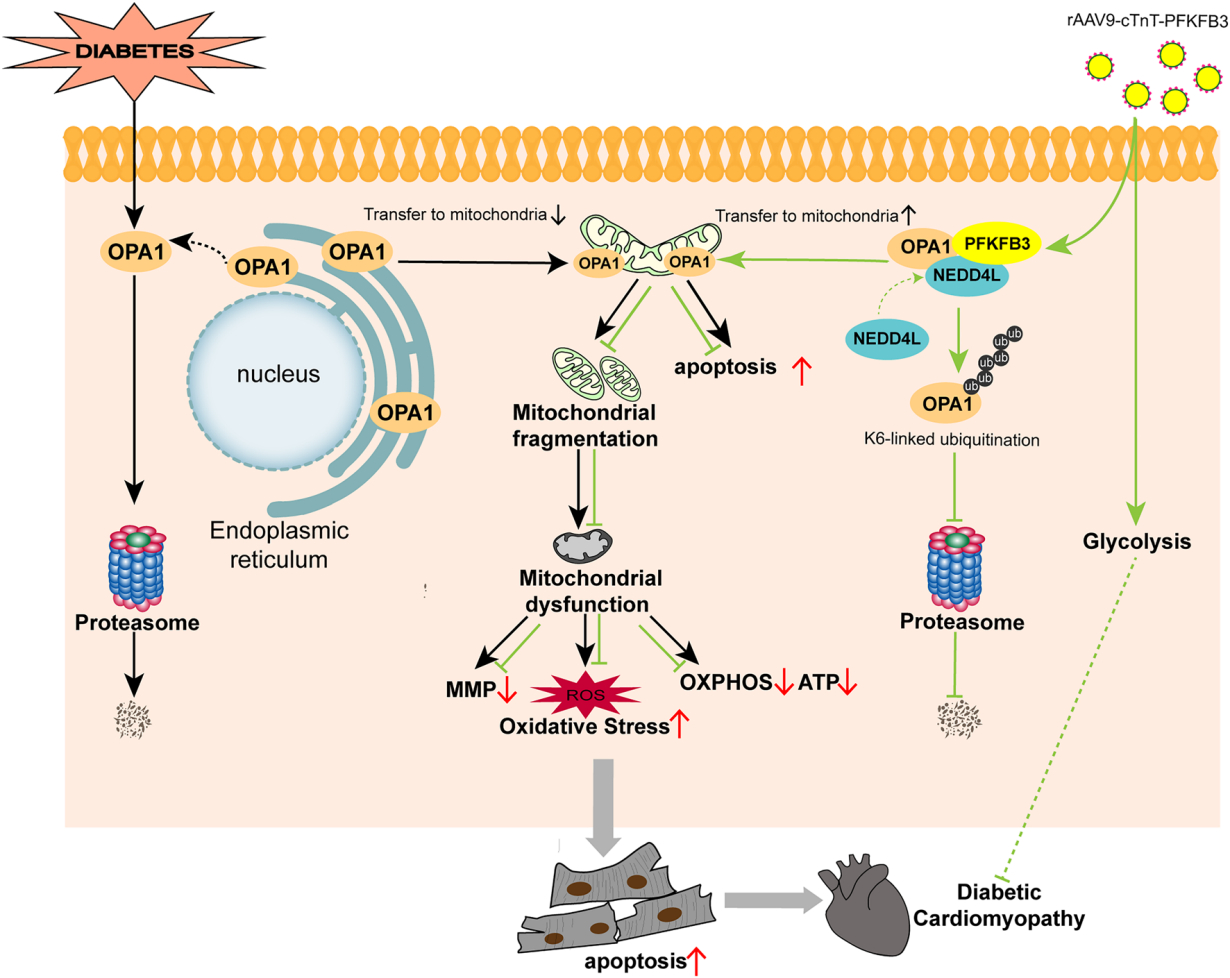

**Supplementary Information:**

The online version contains supplementary material available at 10.1007/s00018-024-05257-5.

## Introduction

Diabetes Mellitus (DM) has become “a pandemic of unprecedented magnitude,” now affecting more than 537 million adults [1]. Cardiovascular disease is a common complication in diabetic patients, causing substantial morbidity and mortality and imposing a significant burden on society [2]. “Diabetic cardiomyopathy” (DCM), defined as impairments in cardiac structure and function without overt hypertension, coronary artery disease (CAD), or valvular disease, has been increasingly regarded as a unique entity since its first definition in 1972 by Rubler et al. [3, 4]. DCM is mainly characterized by left ventricular enlargement, myocardial hypertrophy, myocardial interstitial fibrosis and cardiac systolic and/or diastolic dysfunction, eventually leading to heart failure (HF) [5]. Indeed, traditional glucose-lowering therapy failed to improve cardiac function or reduce the risk of HF, while current routine HF treatment is similar whether or not the patient has DM. Although the emergence of sodium–glucose cotransporter 2 inhibitors (SGLT2i) [6] and glucagon-like peptide 1 receptor antagonists (GLP-1RA) [7] offers some promise in this field, the prevalence of HF in diabetic individuals is still as high as 22%, and the incidence is still rising [8]. Therefore, exploring effective therapeutic strategies for DCM is still the focus of current research.

Mitochondrial dysfunction is implicated as a critical pathogenesis of DCM [9]. The heart is a highly energy-demanding tissue and mitochondria are considered as the “powerhouse” due to their role in providing ATP through oxidative phosphorylation (OXPHOS). Furthermore, mitochondria play an important part in several cellular processes, such as reactive oxygen species (ROS) mediated signaling pathways [10], cytoplasmic calcium homeostasis [11] and apoptosis [12]. Accumulating evidence suggests that mitochondrial dysfunction, characterized by reduced ATP production, impaired oxidative-respiratory chain function, increased ROS production and cardiomyocyte apoptosis participates in the progression of DCM [13, 14]. Hence, focusing on mitochondrial dysfunction may hold promise for preventing DCM.

PFKFB3, also termed 6-phosphofructo-2-kinase/fructose-2,6-biphosphatase 3, assumes a critical function in the generation of stable cytoplasmic concentrations of fructose-2,6-bisphosphate (F-2,6-BP) in glycolysis. This metabolite serves as a preeminent allosteric activator, significantly augmenting the activity of the pivotal regulatory enzyme of glycolysis, phosphofructokinase-1 (PFK-1) [15]. Upregulating the expression of PFKFB3 has been shown to alleviate ROS accumulation and mitochondria dysfunction in human proximal tubular epithelial cells (PTECs), thereby attenuating kidney injury [16]. However, whether PFKFB3 is involved in DCM remains unclear. This study aims to explore the potential effects of PFKFB3 in DCM and the molecular mechanisms involved.

## Methods

### Animals and ethical approval

8-week-old male leptin receptor-deficient (C57BLKS/J-LepRdb/LepRdb, db/db) mice and their control littermates (C57BLKS/J-LepRdb/ +, db/m) were fed under controlled conditions (12-h light/dark cycle, 22 ± 1℃, and 50 ± 10% humidity), with standard chow and water provided ad libitum.

### Statistical analysis

The data is presented as mean ± SD, and statistical analysis was carried out using an unpaired Student's t-test to compare the two groups. We employed one-way ANOVA for multiple groups, followed by Tukey's post hoc analysis. All analyses were performed with GraphPad Prism version 8.0.2 (GraphPad Software, La Jolla, CA). Statistical significance was expressed as p < 0.05.

Extended information about the Materials and Methods can be accessed in the Supplementary Materials.

## Results

### Characterization of PFKFB3 expression in the cardiac tissues and cardiomyocytes

**Fig. 1:**
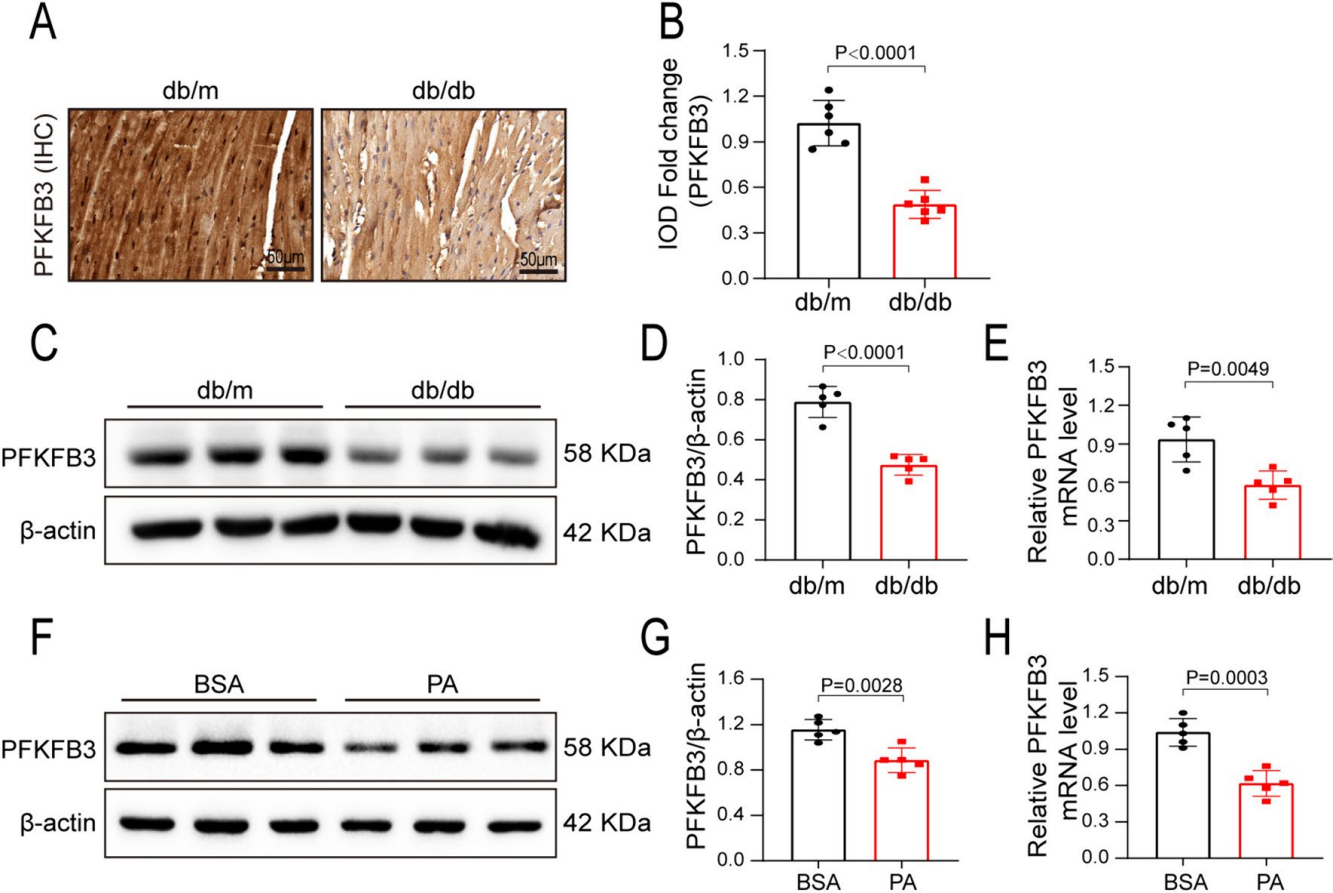
Characterization of PFKFB3 expression in the cardiac tissues and cardiomyocytes. **A** and **B** Representative immunohistochemical staining and quantitative analysis of PFKFB3 expression in the hearts of db/db mice and db/m mice at 20 weeks. IOD, integrated optical density. **C**, **D** Representative Western blot and quantitative analysis of PFKFB3 expression in hearts of 20-week-old db/db mice and db/m mice. **E** The rt-qPCR analysis of PFKFB3 mRNA expression in hearts of 20-week-old db/db mice and db/m mice. n = 5–6/group. **F** and **G** Representative Western blot and quantitation analysis of PFKFB3 expression in AC16 cells treated with BSA or PA for 48 h. **H** The rt-qPCR analysis of PFKFB3 in AC16 cells treated with BSA or PA for 48 h. n = 5/group. Data are expressed as mean ± standard deviation (SD). p < 0.05 was considered significant

We assessed the expression of PFKFB3 in the hearts of db/db and db/m mice. Immunohistochemical staining and Western blot analysis revealed that PFKFB3 expression was considerably reduced in the myocardial tissue of db/db mice compared to db/m mice (Fig. 1A–D). PFKFB3 mRNA level was also reduced in the hearts of db/db animals (Fig. 1E). We also assessed the expression of PFKFB2 in the cardiac tissue. It is an isoenzyme of PFKFB3, while other isoenzymes, such as PFKFB1 and PFKFB4, are not expressed in the heart [17]. The expression of PFKFB2 remained unaltered in the hearts of db/db mice as compared to db/m mice (Fig. S1A, S1B). In vitro, the AC16 human cardiomyocyte cell line was treated with saturated PA (Palmitate acid, 200 μM) or BSA (Bovine serum albumin, solvent control, 10%) for 48 h as previously described [18, 19]. PFKFB3 expression in the protein and mRNA levels was decreased under PA treatment (Fig. 1F–H). These data demonstrated that myocardial PFKFB3 expression was decreased in db/db mice, suggesting a potential involvement in the development of DCM.

### Cardiac-specific overexpression of PFKFB3 alleviated cardiac dysfunction and myocardial remodeling in db/db mice

**Fig. 2:**
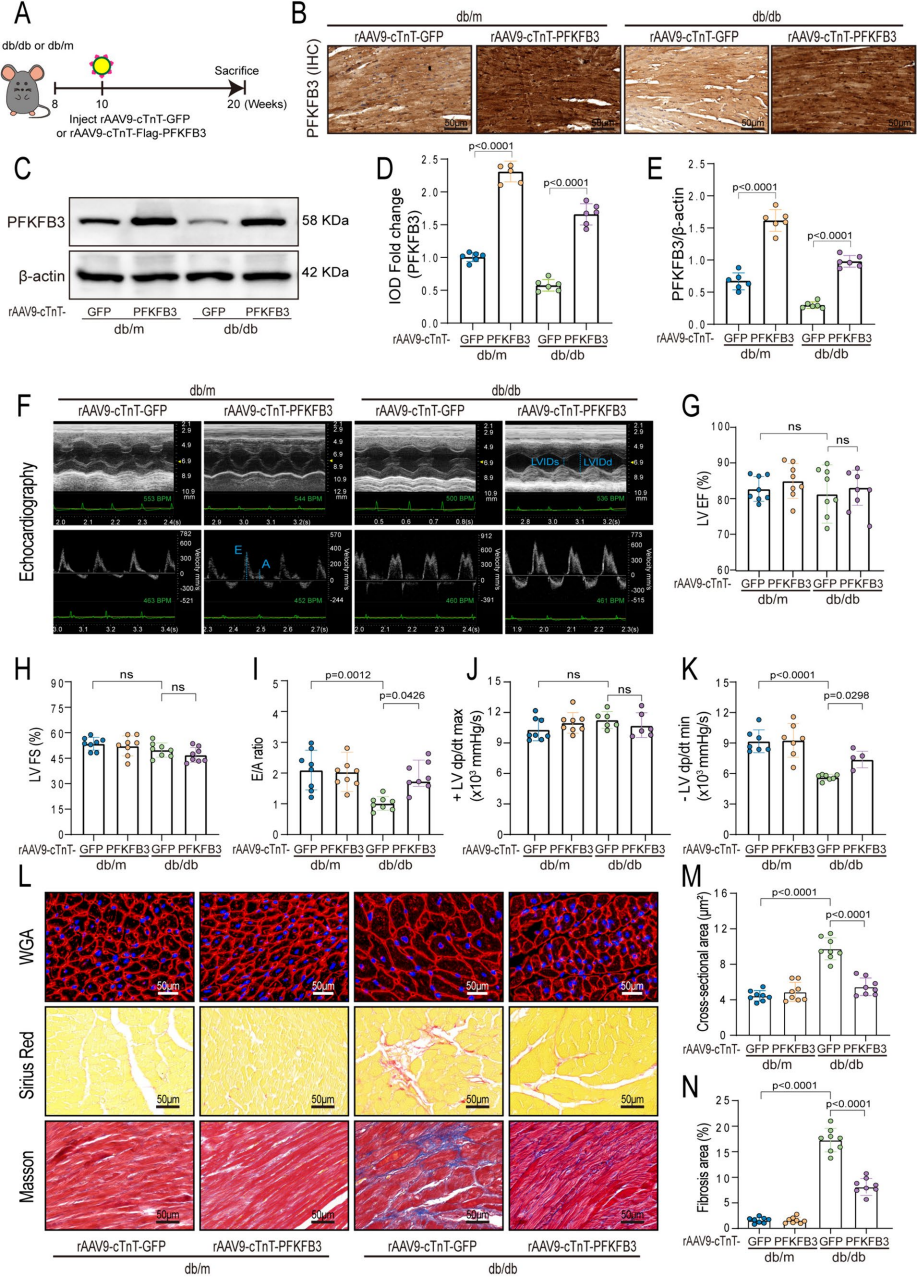
Cardiac-specific overexpression of PFKFB3 alleviated cardiac dysfunction and myocardial remodeling in db/db mice. **A** Schematic diagram. The rAAV9-cTnT-GFP or rAAV9-cTnT-Flag-PFKFB3 were injected via the mice’s tail vein in a volume of 150 μL (5*10^12 vg/mL). **B**, **D.** Representative immunohistochemical staining and quantitative analysis of PFKFB3 in the hearts of db/m and db/db mice. Scale bars, 50 μm. **C**, **E**. Western blot and quantitative analysis of PFKFB3 expression in hearts of db/db and db/m mice. **F** Representative echocardiography images in db/m and db/db mice; LVID: left ventricular internal dimension; s: end-systole; d: end-diastole. **G**–**I** LVEF (left ventricular ejection fraction), LVFS (left ventricular fractional shortening), E/A ratio (the ratio of E wave to A wave). **J**, **K** Hemodynamics analysis of the maximal and minimal first derivative of LV pressure. The heart rate is reliably within the 450–500 bpm range. **L.** Representative images of wheat germ agglutinin (WGA) staining, Sirius Red staining and Masson staining. Scale bars, 50 μm. **M** Quantitative analysis of cardiomyocyte sizes based on WGA staining. **N** Quantitative analysis of fibrosis percentage based on Masson staining. n = 6–8/group. Data are expressed as mean ± SD. p < 0.05 was considered significant

In a prior study, recombinant adeno-associated virus 9 (rAAV9)-mediated gene expression, guided by the cTnT (cardiomyocyte-specific troponin T) promoter, exhibited remarkable cardiac tissue affinity, surpassing the liver by 640-fold [20]. In exploring PFKFB3 overexpression's potential effects on DCM, Ten-week-old mice were injected either with rAAV9-cTnT-flag-PFKFB3 or rAAV9-cTnT-GFP (Fig. 2A). After ten weeks, PFKFB3 was successfully overexpressed in the myocardial tissue of the mice (Fig. 2B–E). In comparison to db/m + rAAV9-cTnT-GFP mice, db/db + rAAV9-cTnT-GFP mice exhibited significant increases in body weight, fasting blood glucose levels, serum total cholesterol (TC) levels, and high-density lipoprotein (HDL) cholesterol levels (Table 1 and Fig. S2A, S2B). Additionally, the db/db + rAAV9-cTnT-GFP mice exhibited impaired glucose tolerance and reduced insulin sensitivity, as verified by the glucose tolerance test (GTT) and insulin tolerance test (ITT) (Fig. S2C–S2F). However, PFKFB3 overexpression had no significant effects on body weight, fasting blood glucose, blood lipids, impaired glucose tolerance, and declined insulin sensitivity in db/db mice. These data indicate that cardiac-specific overexpression of PFKFB3 had no discernible effects on the glycolipid metabolic disorder in db/db mice.

**Table 1:** Blood lipids characteristics in db/m and db/db mice with different adeno-associated virus interventions

Parameter/Group	db/m		db/db	
	rAAV-GFP	rAAV-PFKFB3	rAAV-GFP	rAAV-PFKFB3
Body weight (g)	27.45 ± 2.36	27.98 ± 1.77	61.04 ± 4.47*	61.29 ± 5.10
TC (mmol/L)	2.12 ± 0.24	1.93 ± 0.25	3.39 ± 0.83*	4.00 ± 0.74
TG (mmol/L)	1.01 ± 0.31	1.14 ± 0.32	1.02 ± 0.43	0.70 ± 0.12
LDL (mmol/L)	0.22 ± 0.13	0.25 ± 0.07	0.19 ± 0.07	0.19 ± 0.09
HDL (mmol/L)	1.14 ± 0.14	0.95 ± 0.20	1.57 ± 0.41*	1.76 ± 0.52

*TG* Triglyceride, *TC* total cholesterol, *HDL* high-density lipoprotein, *LDL* low-density lipoprotein

n = 8/group, Data are presented as mean ± SD. *p < 0.05 versus db/m + rAAV9-cTnT-GFP

Echocardiography and Millar catheter were utilized to assess cardiac function and hemodynamic parameters. No significant alterations in fractional shortening (FS), ejection fraction (EF) and + LV dp/dt max (maximal first derivative of left ventricular pressure over time; a measure of cardiac contractility) between the db/m + rAAV9-cTnT-GFP and the db/db + rAAV9-cTnT-GFP mice (Fig. 2F–H, J). The E/A ratio (the ratio of early (E-wave) to late (A-wave) mitral inflow velocity) and -LV dp/dt min (minimal first derivative of left ventricular pressure over time; a measure of cardiac relaxation) were decreased in the db/db + rAAV9-cTnT-GFP mice compared to the db/m + rAAV9-cTnT-GFP mice, but reversed by PFKFB3 overexpression (Fig. 2F, I, K). Cardiac hypertrophy and interstitial fibrosis are pivotal alterations in DCM. Compared with db/m + rAAV9-cTnT-GFP mice, left ventricular mass was increased in db/db + rAAV9-cTnT-GFP mice, which was mitigated by PFKFB3 overexpression (Table 2). Additionally, db/db + rAAV9-cTnT-GFP mice displayed evident cardiac remodeling, characterized by enlarged cardiomyocyte cross-sectional areas and heightened interstitial collagen deposition within the myocardium, which was alleviated by PFKFB3 overexpression (Fig. 2L–N). In conclusion, cardiac-specific overexpression of PFKFB3 significantly improved cardiac function and attenuated myocardial remodeling in db/db mice.

**Table 2:** Echocardiographic characteristics in db/m and db/db mice with different adeno-associated virus interventions

Parameter/Group	db/m		db/db	
	rAAV-GFP	rAAV-PFKFB3	rAAV-GFP	rAAV-PFKFB3
E/A	2.14 ± 0.62	2.08 ± 0.61	1.04 ± 0.21*	1.77 ± 0.41#
LVEF (%)	81.38 ± 3.02	83.44 ± 4.24	80.04 ± 7.11	81.71 ± 4.29
LVFS (%)	54.08 ± 3.57	51.53 ± 6.13	50.34 ± 3.97	49.92 ± 8.45
SV (μL)	31.08 ± 1.99	31.54 ± 5.25	31.35 ± 4.67	31.59 ± 5.83
HR (b.p.m)	523 ± 49	534 ± 33	527 ± 42	511 ± 26
CO (mL/min)	17.49 ± 1.38	17.80 ± 2.36	20.80 ± 5.01	17.02 ± 8.12
LV Mass (mg)	133.06 ± 21.06	133.57 ± 22.18	168.01 ± 9.55*	136.93 ± 23.71#
LVAW; s (mm)	1.74 ± 0.12	1.75 ± 0.16	1.80 ± 0.25	1.75 ± 0.19
LVAW; d (mm)	1.09 ± 0.14	1.02 ± 0.22	1.12 ± 0.19	1.03 ± 0.24
LVPW; s (mm)	1.59 ± 0.26	1.61 ± 0.28	1.58 ± 0.35	1.63 ± 0.14
LVPW; d (mm)	1.09 ± 0.18	1.17 ± 0.26	1.06 ± 0.39	1.11 ± 0.22
LVID; s (mm)	1.19 ± 0.30	1.15 ± 0.26	1.27 ± 0.58	1.17 ± 0.33
LVID; d (mm)	2.92 ± 0.24	2.86 ± 0.30	3.25 ± 0.29	2.79 ± 0.54

*E/A* ratio of E wave to A wave, *LVEF* left ventricle ejection fraction, *LV*FS left ventricle fractional shortening, *SV* stroke volume, *HR* heart rate, CO cardiac output, *LV Mass* left ventricle mass, *LVAW,*s left ventricular anterior wall thickness at end-systole, *LVAW,d* left ventricular anterior wall thickness at end-diastole, *LVPW,s* left ventricular posterior wall thickness at end-systole, *LVPW,d* left ventricular posterior wall thickness at end-diastole, *LVID,s* left ventricular internal-systolic dimension, *LVID,d* left ventricular internal-diastolic dimension. n = 8/group

Data are presented as mean ± SD, *p < 0.05 versus db/m + rAAV9-cTnT-GFP, #p < 0.05 versus db/db + rAAV9-cTnT-GFP

### Cardiac-specific overexpression of PFKFB3 inhibited myocardial oxidative stress and apoptosis

**Fig. 3:**
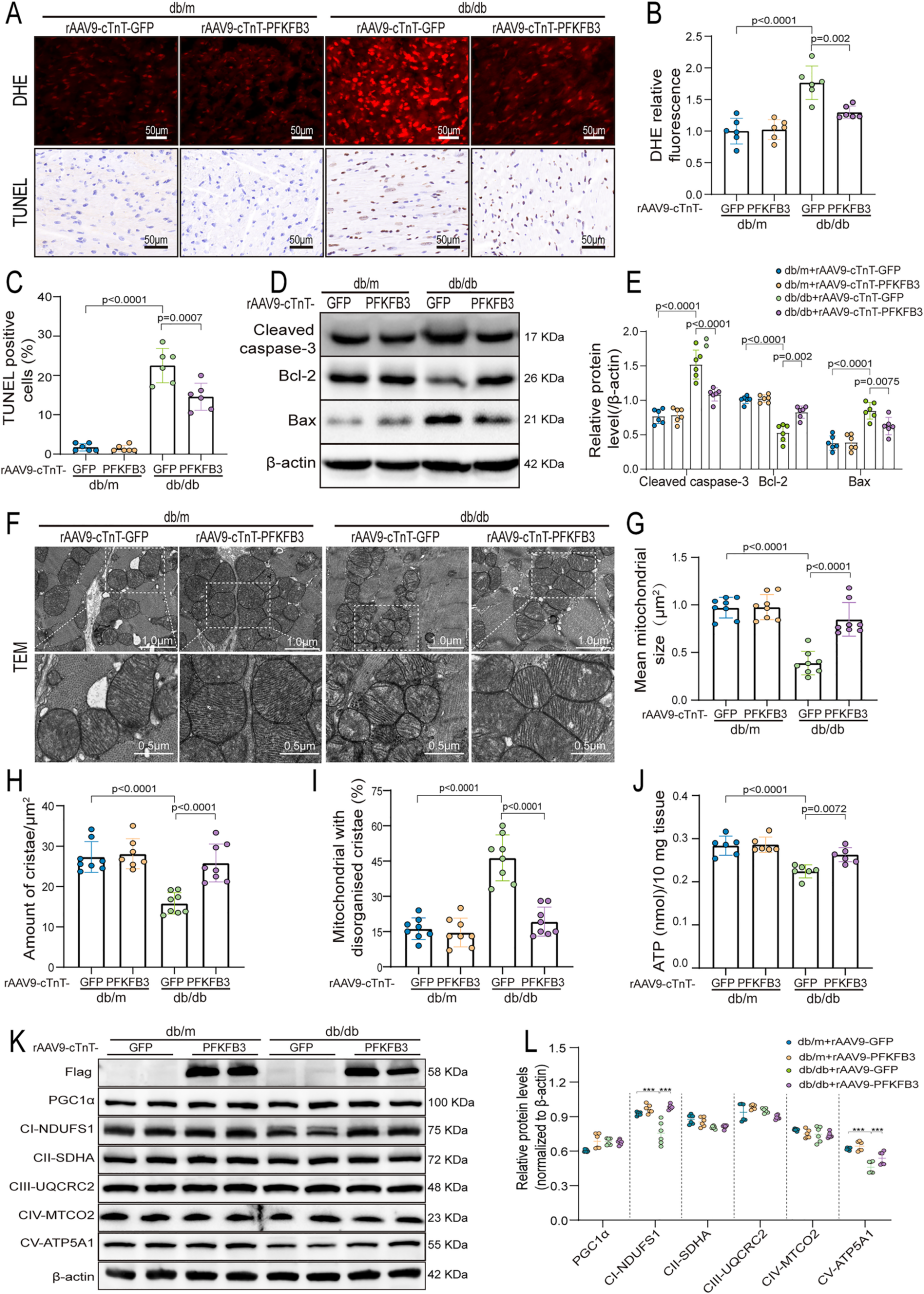
Cardiac-specific overexpression of PFKFB3 inhibited myocardial oxidative stress, apoptosis, mitochondrial fragmentation and dysfunction in db/db mice. **A** Representative DHE and TUNEL staining images. Scale bars, 50 μm. **B** Quantitative analysis of DHE relative fluorescence. **C** Quantitative data of the TUNEL positive cells. **D**, **E** Representative Western blot of Cleaved caspase-3, Bax, Bcl-2 and associated quantitation analysis. **F** Representative transmission electron microscopic images of mitochondria. Scale bars, 1.0 μm, 0.5 μm. **G** Quantitative analysis of mean mitochondrial size. **H** The cristae number. **I** The proportion of mitochondria with disorganized cristae. **J** Quantification of ATP levels per 10 mg cardiac tissue. **K**, **L** Representative Western blot and quantitative analysis of Flag, PGC-1α and mitochondrial respiratory chain complex I–V (CI–CV). n = 6–8/group. Data are expressed as mean ± SD. p < 0.05 was considered significant

Previous evidence suggests that increased oxidative stress and cardiomyocyte apoptosis are prevalent characteristics of DCM [21, 22]. As expected, the apoptosis rate determined by TUNEL staining (Fig. 3A, C) and the levels of apoptosis-related proteins, including Bax and Cleaved caspase-3, were elevated in myocardial tissues of db/db + rAAV9-cTnT-GFP mice compared to db/m + rAAV9-cTnT-GFP mice (Fig. 3D, E). In contrast, the level of the anti-apoptosis protein Bcl-2 was lower in db/db + rAAV9-cTnT-GFP mice (Fig. 3D, E). Additionally, the amount of myocardial superoxide anion was increased in db/db + rAAV9-cTnT-GFP mice compared to that in the db/m + rAAV9-cTnT-GFP group, as determined by dihydroethidium (DHE) staining (Fig. 3A, B). In db/db mice, the overexpression of PFKFB3 in the myocardium led to a significant reduction in cardiomyocyte apoptosis and oxidative stress (Fig. 3A–E).

In vitro, we transfected AC16 cells with pcDNA3.1 or pcDNA3.1-Flag-PFKFB3 plasmids. The overexpression of PFKFB3 significantly reduced apoptosis in PA-treated AC16 cells, as evidenced by the decrease in the percentage of apoptotic cells (Fig. S3A, S3D), the decreased expression of Bax and Cleaved caspase-3 coupled with elevated levels of Bcl-2 (Fig. S3C, S3F). Similarly, flow cytometry analysis revealed that PFKFB3 overexpression significantly decreased ROS levels induced by PA in AC16 cells (Fig. S3B, S3E). In summary, cardiac-specific overexpression of PFKFB3 significantly inhibited myocardial oxidative stress and apoptosis in DCM.

### Cardiac-specific overexpression of PFKFB3 reduced mitochondrial fragmentation and partially restored mitochondrial function

Impaired mitochondrial function increases ROS production and apoptosis in cardiovascular disease [23]. Abnormal-shaped mitochondria, characterized by a reduction of the mean size, were observed in myocardial samples from db/db + rAAV9-cTnT-GFP mice compared to db/m + rAAV9-cTnT-GFP mice using transmission electron microscopy (TEM) (Fig. 3F and G). Additionally, a substantial loss of electron density and decreased mitochondrial cristae, accompanied by an increased proportion of ruptured and disorganized cristae, were observed in diabetic hearts compared to control mice (Fig. 3F, H, I). Similarly, MitoTracker staining of PA-treated AC16 cells revealed mitochondrial fragmentation (Fig. S4A and S4B). However, PFKFB3 overexpression promoted mitochondrial fusion, as evidenced by increased mitochondrial size in the hearts of db/db mice and partially recovery of the mitochondrial network in PA-treated AC16 cells (Figs. 3F and G, S4A and S4B). The mitochondrial membrane potential (MMP) was measured using the JC-1 dye and a flow cytometer. The ratio of monomers that produced green fluorescence indicated the degree of mitochondrial depolarization. PA treatment triggered heightened mitochondrial depolarization, resulting in a reduction in MMP, which was inhibited by PFKFB3 overexpression (Fig. S4C and S4D).

Since the mitochondria serve as the epicenters of OXPHOS and ATP synthesis in mammalian cells, we scrutinized mitochondrial respiratory capacity by assessing mitochondrial oxygen consumption rate (OCR) and ATP levels. As predicted, PA treatment significantly reduced mitochondrial respiratory capacity, including basal respiration, ATP-related respiration, and maximal respiration, compared to pcDNA3.1-treated AC16 cells (Fig. S4E and S4F). Concurrently, ATP levels were decreased in the hearts of db/db + rAAV9-cTnT-GFP mice compared to db/m + rAAV9-cTnT-GFP mice (Fig. 3J). Remarkably, PFKFB3 overexpression significantly enhanced mitochondrial respiration and increased ATP levels (Figs. 3J and S4E and S4F). Moreover, we assessed the level of mitochondrial biogenesis-related protein PGC-1α and the mitochondrial respiratory chain complexes I–V, designated as CI-NDUFS1, CII-SDHA, CIII-UQCRC2, CIV-MTCO2, and CV-ATP5A1. The presence of the Flag validated the efficiency of PFKFB3 overexpression (Fig. 3K). There were no noteworthy distinctions in PGC-1α and mitochondrial complexes II, III, and IV across the experimental groups. However, in the hearts of db/db + rAAV9-cTnT-GFP mice, the expression of mitochondrial complexes I and V was markedly decreased, which was partially recovered by PFKFB3 overexpression (Fig. 3K and L). The findings highlight the crucial role of PFKFB3 overexpression in preserving mitochondrial integrity and alleviating mitochondrial dysfunction in db/db myocardial tissue.

Impaired glycolysis has been observed in the cardiac tissues of both diabetic individuals and experimental diabetic animals [24], and several studies have reported that restoring glycolytic metabolism alleviated DCM [25–27]. PFKFB3 is a regulator of glycolytic activity [28–30]. Li et al. discovered that elevating SIRT3 levels relieves DCM by upregulating the expression of PFKFB3, thereby enhancing glycolytic processes. So, we analyzed lactic acid levels (a product of glycolysis) in the cardiac tissues of mice and assessed the extracellular acidification rate (ECAR) in AC16 cells. As expected, PFKFB3 overexpression resulted in elevated lactic acid levels in the hearts of both db/m and db/db mice (Fig. S5A). In AC16 cells, PFKFB3 overexpression increased baseline ECAR, glycolysis, and glycolytic capacity, regardless of PA intervention (Fig. S5B, S5C). Consistent with Li's study, our results suggest that PFKFB3 overexpression stimulated glycolysis. 2-DG (2-deoxyglucose, a hexokinase 2 inhibitor) is a common glycolytic inhibitor that significantly inhibits the level of glycolysis (Fig. S5B). Interestingly, under the condition of inhibited glycolysis with 2-DG, overexpression of PFKFB3 still suppressed PA-induced oxidative stress and apoptosis, which indicates that PFKFB3 overexpression can alleviate DCM independent of glycolysis (Fig. S5D–S5G).

### PFKFB3 interacts with OPA1

**Fig. 4:**
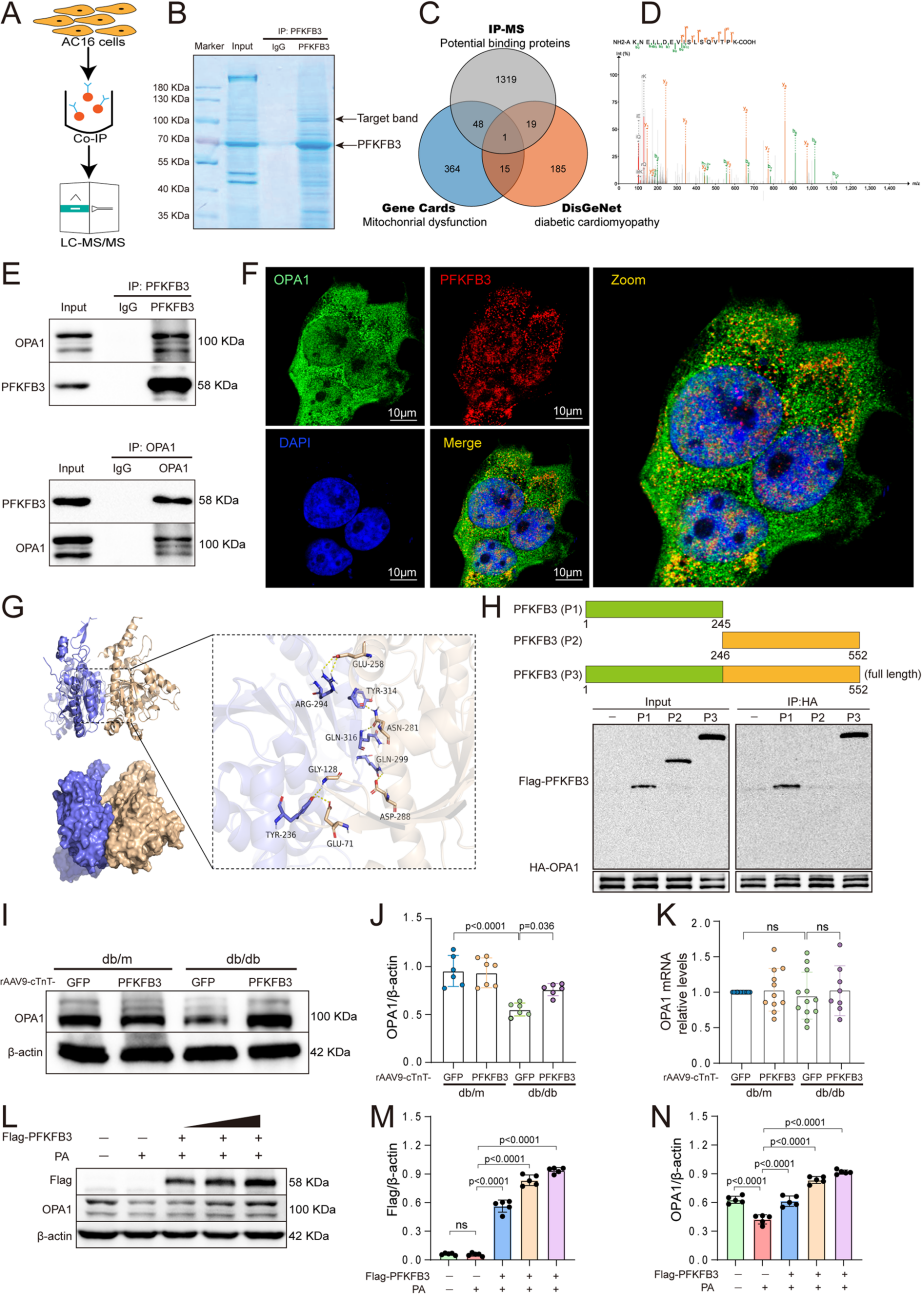
PFKFB3 interacts with OPA1. **A** Schematic diagram. **B** Coomassie Blue staining. **C** Venn diagram. **D** Mass spectrogram of OPA1. **E** Endogenous immunoprecipitation of PFKFB3 and OPA1 in cardiac tissue. **F** Immunofluorescence of PFKFB3 (red) and OPA1 (green) in AC16 cells, scale bars, 10 μm. **G** The interaction diagram of PFKFB3 protein and OPA1 protein shows that the wheat color is OPA1, the blue color is PFKFB3, and the yellow dotted line indicates hydrogen bonding. **H** Co-IP assay of PFKFB3 deletion mutants and HA-OPA1. HEK293T cells were co-transfected with HA-OPA1 and either pcDNA3.1 or Flag-PFKFB3 1–245(P1) or Flag-PFKFB3 246–552(P2) or Flag-PFKFB3 full-length (P3).** I**, **J** Representative Western blot and quantitative analysis of OPA1 expression in db/m and db/db mice. **K** The rt-qPCR analysis of OPA1 mRNA expression in db/m and db/db mice. **L–N** Representative Western blot and quantitative analysis of OPA1 and Flag expression under the treatment of PA and transfected with Flag-PFKFB3 plasmids in different doses. n = 6–8/group. Data are expressed as mean ± SD. p < 0.05 was considered significant

To investigate the mechanisms underlying the protective effects of PFKFB3 overexpression against DCM other than glycolysis, we employed mass spectrometry. AC16 cells were exposed to adenovirus-PFKFB3 (Ad-PFKFB3) or control vectors (Ad-GFP) for 12 h, followed by a 48-h treatment with PA. Subsequently, immunoprecipitation and immunoblotting analyses were performed, accompanied by Coomassie Blue Staining and liquid chromatography-tandem mass spectrometry (LC–MS/MS) (Fig. 4A, B). Next, we compared the 1387 potential copurified proteins with the 220 DCM-related proteins from DisGeNet (a database of human gene-disease associations) and the 428 mitochondrial dysfunction-related proteins from GeneCards (The Human Gene Database) (Fig. 4C). Only one potential binding protein, optic atrophy 1 (OPA1), was selected. The mass spectrogram of OPA1 is demonstrated in Fig. 4D. To validate the interaction between PFKFB3 and OPA1 in DCM, we conducted co-immunoprecipitation (Co-IP) assays using antibodies against PFKFB3 and OPA1 in the hearts of db/db mice (Fig. 4E). Additionally, we conducted immunofluorescence staining to visually demonstrate the colocalization of PFKFB3 and OPA1 in AC16 cells. The images revealed a robust colocalization between PFKFB3 and OPA1 (Fig. 4F).

By employing ZDOCK and the Prodigy server for protein–protein docking, we could anticipate the binding site and the affinity of PFKFB3 towards OPA1, calculated at −11.3 kcal/mol (Fig. 4G). To identify the specific binding motif of PFKFB3, we introduced two truncated mutations of PFKFB3, spanning amino acids 1–245 (P1) and 246–552 (P2). HEK293T cells were transfected with HA-OPA1 and either pcDNA3.1 or Flag-PFKFB3 1–245 (P1) or Flag-PFKFB3 246–552 (P2) or full-length Flag-PFKFB3 (P3), followed by Co-IP analysis. As indicated in Fig. 4H, both full-length Flag-PFKFB3 (P3) and Flag-PFKFB3 1–245 (P1) effectively immunoprecipitated with HA-OPA1, but Flag-PFKFB3 246–552 (P2) failed to immunoprecipitate with HA-OPA1. This implies that PFKFB3 interacted with OPA1 via its N-terminal domain (amino acids 1–245).

The interaction between PFKFB3 and OPA1 raises an intriguing question: Does PFKFB3 modulate OPA1? Our data reveal a significant reduction in OPA1 expression in the hearts of db/db mice compared to db/m mice. Notably, PFKFB3 overexpression partially restored OPA1 protein levels in the hearts of db/db mice, while mRNA levels of OPA1 remained unaffected (Fig. 4I–K). In vitro, OPA1 expression decreased in PA-treated AC16 cells. However, the transfection of AC16 cells with Flag-PFKFB3-encoding plasmids effectively rescued this decline in OPA1, increasing its protein levels in a dose-dependent manner (Fig. 4L–N). These data robustly indicate that PFKFB3 interacts with and regulates the protein levels of OPA1.

### OPA1 knockdown attenuated the effects of PFKFB3 overexpression in alleviating cardiac dysfunction and remodeling in db/db mice

**Fig. 5:**
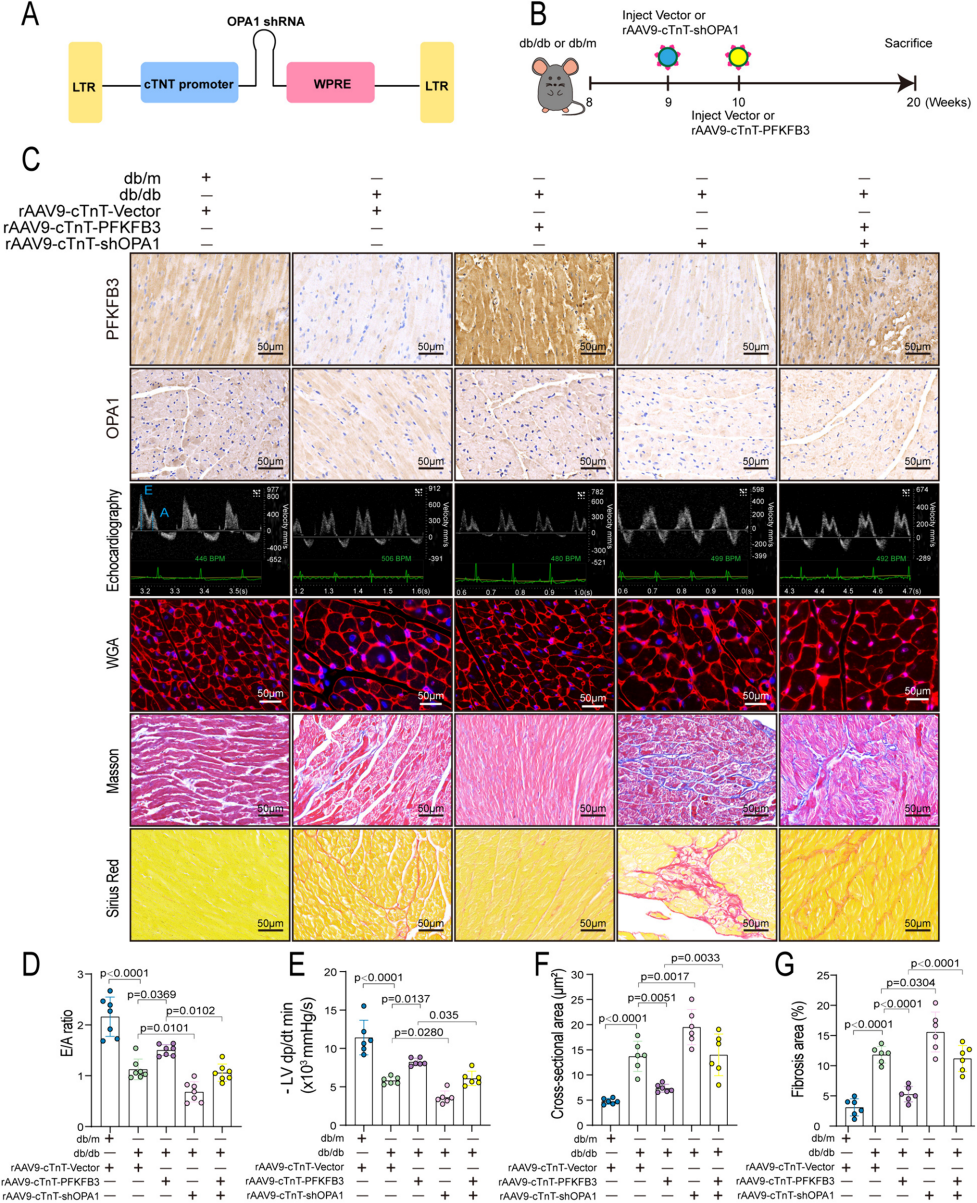
OPA1 knockdown attenuated the effects of PFKFB3 overexpression in alleviating cardiac dysfunction and remodeling in db/db mice. **A** Schematic diagram of AAV9-cTnT-shOPA1 structure. **B** Schedule of animal experiments. Nine-week-old mice were injected with rAAV9-cTnT-shOPA1 or vector, and then a week later, mice were injected with rAAV9-cTnT-Flag-PFKFB3 or vector. Finally, mice were sacrificed at the age of 20 weeks. **C** Immunohistochemical staining of PFKFB3 and OPA1 expression. Echocardiography displays the E/A ratio. WGA staining was used to evaluate cardiomyocyte size. Masson staining and Sirius Red staining were used to assess fibrosis. **D** Quantitative analysis of E/A ratio. **E** Haemodynamic analysis of the minimal first derivative of LV pressure (-LV dP/dt min). **F** Quantitative analysis of WGA staining for cross-section area. **G** Quantitative analysis of Masson staining for fibrosis area. n = 6/group. Data are expressed as mean ± SD. p < 0.05 was considered significant

To uncover OPA1’s pivotal role in mediating PFKFB3’s protection against DCM, we utilized a cardiomyocyte-specific viral shRNA targeting OPA1, driven by the cTnT promoter (Fig. 5A). The procedure is outlined in Fig. 5B. Briefly, nine-week-old db/db mice received either rAAV9-cTnT-shOPA1 or rAAV9-cTnT-vector. One week later, these mice received injections with rAAV9-cTnT-Flag-PFKFB3 or rAAV9-cTnT-vector. Immunohistochemistry and Western blot confirmed OPA1 knockdown and PFKFB3 overexpression. rAAV9-cTnT-shOPA1 significantly reduced OPA1 expression while rAAV9-cTnT-flag-PFKFB3 markedly increased PFKFB3 levels in the myocardium of db/db mice (Fig. 5C, Fig. S6A–S6F). The echocardiographic and hemodynamic analysis demonstrated that cardiomyocyte-specific overexpression of PFKFB3 inhibited the decline in diastolic function, as evidenced by the elevated E/A ratio and -LV dp/dt min in diabetic hearts. However, the knockdown of OPA1 blocked the protective effects of PFKFB3 on diastolic function in db/db mice (Fig. 5C–E). Moreover, PFKFB3 overexpression attenuated cardiac remodeling, including cardiomyocyte hypertrophy and myocardial interstitial fibrosis, while OPA1 knockdown in cardiomyocytes nullified PFKFB3’s protective effects in diabetic hearts (Fig. 5C, F and G). Thus, cardiomyocyte-specific OPA1 knockdown significantly attenuated the beneficial impact of PFKFB3 on cardiac structure and function, underscoring the PFKFB3’s protective effect against DCM depending on the presence of OPA1.

### OPA1 knockdown attenuated the inhibitory effects of PFKFB3 overexpression on myocardial apoptosis and oxidative stress

**Fig. 6:**
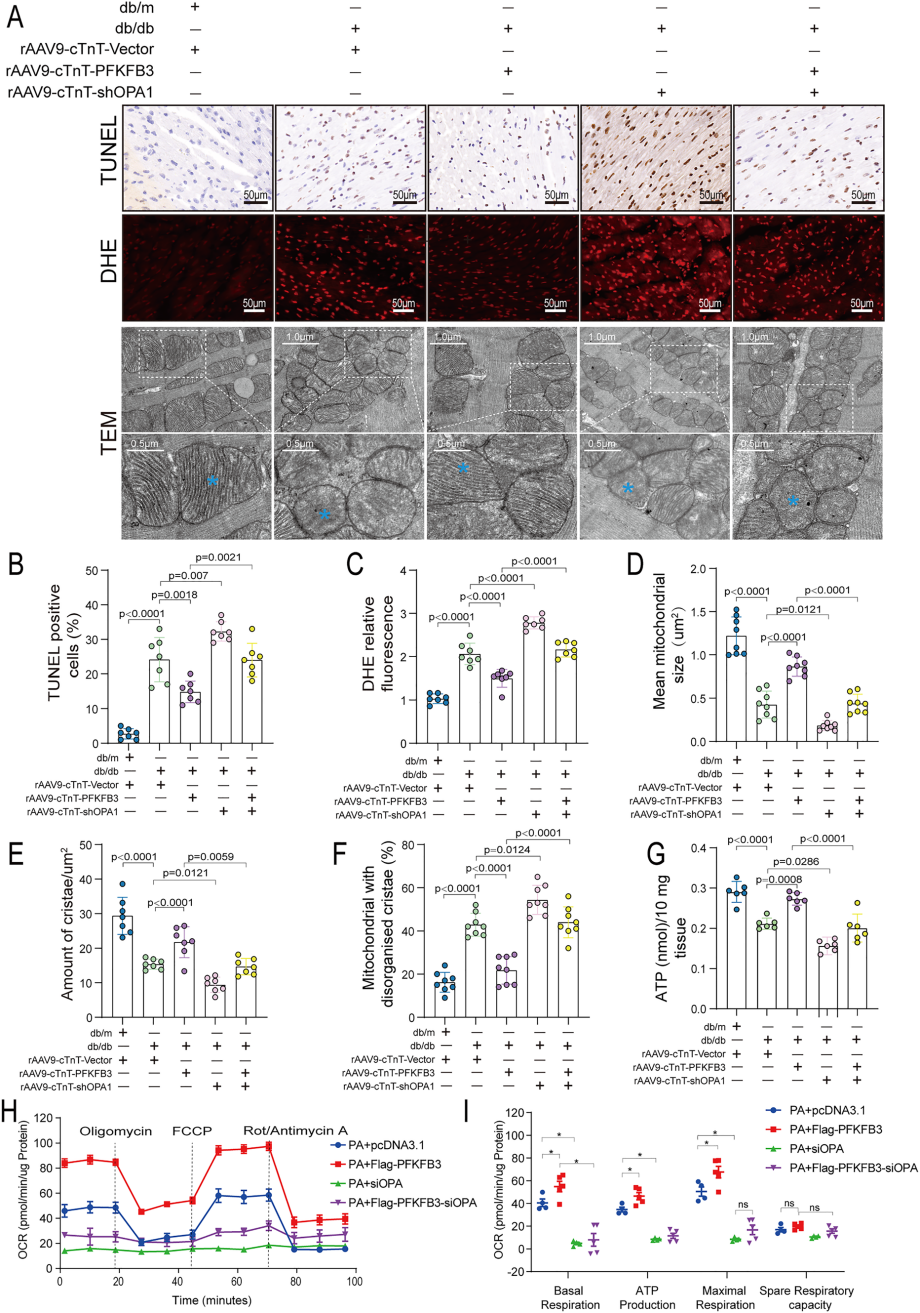
OPA1 knockdown attenuated the inhibitory effects of PFKFB3 overexpression on myocardial oxidative stress, apoptosis, mitochondrial fragmentation, and dysfunction in db/db mice. **A** Representative TUNEL staining images (Scale bars, 50 μm), DHE staining microphotographs (Scale bars, 50 μm) and transmission electron microscopic images of mitochondria (Scale bars, 1 μm, 0.5 μm). **B** Quantitative data of the percentage of TUNEL positive cells. **C** Quantitative analysis of DHE relative fluorescence. **D** Quantitative analysis of mean mitochondrial size. **E** Cristae number per μm^2^. **F** The proportion of mitochondria with disorganized cristae. **G** Quantification of ATP levels per 10 mg cardiac tissue. n = 6–7/group. **H**, **I** Oxygen consumption rate (OCR) and quantitative statistical analysis of OCR, n = 4/group. Data are expressed as mean ± SD. p < 0.05 was considered significant

OPA1 has been reported to protect cells from apoptosis and inhibit oxidative stress [31–33]. Therefore, we further explored whether PFKFB3 overexpression improves cardiomyocyte apoptosis and oxidative stress in db/db mice through OPA1. As anticipated, the TUNEL staining indicated that cardiomyocyte-specific overexpression of PFKFB3 significantly reduced the percentage of apoptotic cardiomyocytes (Fig. 6A and B). DHE staining demonstrated that PFKFB3 overexpression effectively inhibited the production of superoxide anion in diabetic hearts (Fig. 6A, C). However, specific knockdown of OPA1 in cardiomyocytes attenuated all the inhibitory effects of PFKFB3 on myocardial apoptosis and oxidative stress in diabetic hearts (Fig. 6A–C). Moreover, flow cytometry results revealed that transfection of Flag-PFKFB3 plasmids significantly alleviated apoptosis induced by PA and the production of ROS in vitro (Fig. S7A–S7D). To further validate whether the beneficial effects of PFKFB3 in anti-apoptosis and anti-oxidative stress depend on OPA1 in vitro, we designed a specific small interfering RNA (siRNA) to knock down the expression of OPA1. Consistent with in vivo findings, OPA1 knockdown using siRNA attenuated the inhibitory effects of PFKFB3 overexpression on PA-induced apoptosis and ROS production (Fig. S7A–S7D). In conclusion, PFKFB3 overexpression inhibited myocardial apoptosis and oxidative stress in db/db mice, dependent on OPA1 expression.

### OPA1 knockdown attenuated the protective effects of PFKFB3 overexpression on mitochondrial morphology and function

Since OPA1 is an essential regulatory protein during mitochondrial fusion, we explored whether PFKFB3 overexpression regulates mitochondrial morphology and function through OPA1. TEM unveiled that PFKFB3 overexpression increased the mean size of mitochondria and the number of cristae, while reducing the number of disorganized cristae (Fig. 6A, D–F). Additionally, MitoTracker staining demonstrated that transfecting Flag-PFKFB3 inhibited the mitochondrial fragmentation induced by PA (Fig. S8A, S8C). However, OPA1 knockdown attenuated the beneficial effects of PFKFB3 overexpression on mitochondrial morphology and ultrastructure (Fig. 6A, D–F). In vitro, OPA1 knockdown using siRNA attenuated the protective effects of PFKFB3 overexpression on the mitochondrial network in AC16 cells treated with PA (Fig. S8A, S8C). Mitochondrial MMP, OXPHOS and ATP production are typical indicators for evaluating mitochondrial function. Consistent with expectations, transfection of Flag-PFKFB3 inhibited the decrease in MMP level induced by PA (Fig. S8B, S8D). Furthermore, PFKFB3 overexpression significantly improved the mitochondrial respiratory capacity in PA-treated AC16 cells, characterized by increased basal respiration, ATP-related respiration and maximal respiration (Fig. 6H, I). PFKFB3 overexpression also raised the ATP levels in diabetic myocardium (Fig. 6G). Nonetheless, the knockdown of OPA1 greatly attenuated the beneficial effects of PFKFB3 overexpression on mitochondrial MMP, ATP production, and respiratory function (Figs. 6G–I, S8B, S8D). In conclusion, these results reveal that PFKFB3 overexpression exerts beneficial effects on mitochondrial morphology and function through an OPA1-dependent mechanism in DCM.

### PFKFB3 stabilizes OPA1 expression by catalyzing K6-linked polyubiquitination through the E3 ubiquitin ligase NEDD4L

**Fig. 7:**
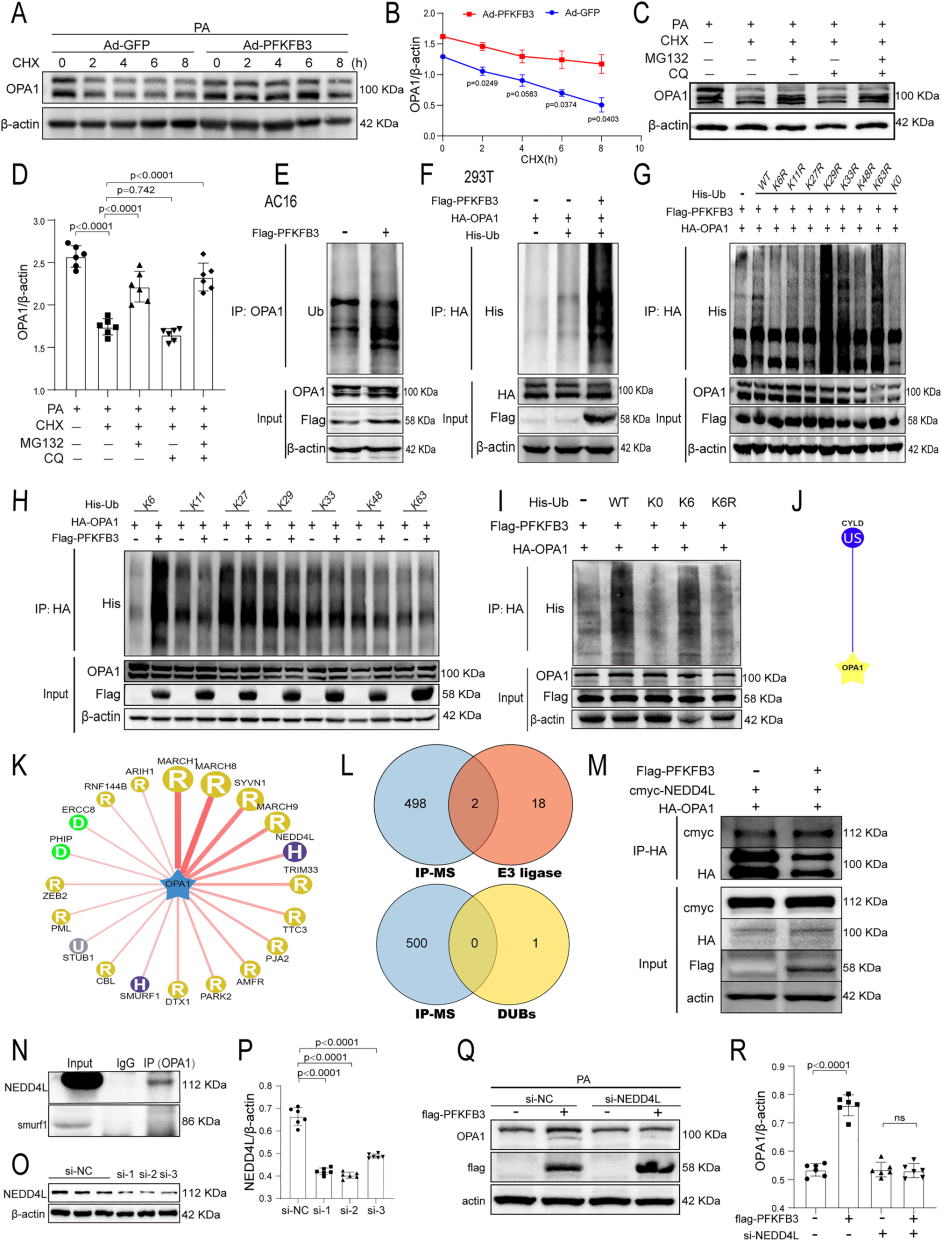
PFKFB3 stabilizes OPA1 by catalyzing K6-linked polyubiquitination through the E3 ubiquitin ligase NEDD4L. **A**, **B**. Western blot and quantification analysis of OPA1 protein. AC16 cells were pretreated with Ad-PFKFB3 and Ad-GFP for 12 h. Following PA (200 μM) treatment for 48 h, cells were exposed to CHX (cycloheximide, 100 μg/ml) for indicated times before harvest. **C**, **D** Western blot and quantification of OPA1 protein in AC16 cells treated with PA (200 μM) for 48 h. CHX (100 μg/ml), MG132 (proteasome inhibitors, 5 μM), and CQ (chloroquine, autophagy lysosomal inhibitors, 20 μM) were administered for 6 h before harvest. **E** Co-Immunoprecipitation (IP) of AC16 cells transfected with Flag-PFKFB3 plasmids or vectors. **F** Co-IP of HEK293T cells transfected with HA-OPA1, Flag-PFKFB3, and His-ubiquitin plasmids. **G**, **H** Influence of ubiquitin KR (Lys to Arg) mutants (**G**) and K-only ubiquitin mutants (**H**) on PFKFB3-mediated OPA1 polyubiquitination. HEK293T cells were transfected with specified constructs, and OPA1 ubiquitination was assessed. **I** Impact of wild type, K0, K6, and K6R on PFKFB3-mediated OPA1 polyubiquitination. **J**, **K** Identification of top 20 predicted E3 ubiquitin ligases (**K**) and the sole deubiquitinase (CYLD) (**J**) using the Ubibrowser website. **L** Venn diagram illustrating the intersection of IP-MS analysis of OPA1 with predicted E3 ubiquitin ligases and deubiquitinase of the OPA1 protein. **M** Co-IP assay of HA-OPA1 and cmyc-NEDD4L in HEK293T cells, with or without Flag-PFKFB3 presence. **N** Immunoprecipitation using an anti-OPA1 antibody, followed by Western blot analysis with anti-NEDD4L and anti-smurf1 antibody, utilizing cellular extracts from AC16 cells. **O** Assessment of NEDD4L small interfering RNA (siRNA) knockdown efficiency. **P** Quantitative analysis of NEDD4L siRNA transfection efficiency. **Q**, **R** Western blot and quantitative analysis of OPA1 in AC16 cells transfected with either negative control siRNA or NEDD4L siRNA for 6 h, followed by transfection with pcDNA3.1 or Flag-PFKFB3 for 6 h, and finally, PA treatment for 48 h. n = 6/group. Data are expressed as mean ± SD. p < 0.05 was considered significant

Given the potential effects of OPA1 in PFKFB3 overexpression-mediated inhibitory action against DCM, we investigated the underlying mechanisms by which PFKFB3 overexpression upregulates OPA1 expression. As shown in Fig. 4K, PFKFB3 overexpression did not affect the OPA1 mRNA level. Therefore, we further explored whether increased OPA1 expression induced by PFKFB3 overexpression was mediated by inhibited degradation at the protein level. We pretreated AC16 cells with Ad-GFP and Ad-PFKFB3, followed by PA treatment for 48 h. Cycloheximide (CHX), a protein synthesis inhibitor, was then added at different time points before harvest. Western blot analysis demonstrated a lower rate of OPA1 degradation in the Ad-PFKFB3 treated cells (Fig. 7A, B). Within eukaryotic cells, two principal mechanisms for protein degradation exist: the autophagy-lysosomal pathway and the ubiquitin-proteasomal pathway. To determine which pathway was involved in OPA1 degradation under PA treatment, we pretreated AC16 cells with Chloroquine (a specific lysosomal inhibitor) and MG132 (a proteasomal inhibitor), either separately or in combination. We used CHX as a positive control in these experiments. Our findings indicated that MG132, either alone or in combination with Chloroquine, significantly blocked the degradation of OPA1 (Fig. 7C and D). The data indicated that PFKFB3 inhibited the degradation of OPA1 via the proteasome pathway.

Modifying a protein with ubiquitin chains is the hallmark of protein degradation by the ubiquitin–proteasome system (UPS). Interestingly, PFKFB3 overexpression increased the endogenous ubiquitination levels of OPA1 (Fig. 7E). To investigate this further, we co-transfected HEK293T cells with plasmids expressing HA-OPA1 and/or Flag-PFKFB3 and/or His-Ubiquitin. The data revealed that PFKFB3 overexpression enhanced the exogenous ubiquitination of OPA1 (Fig. 7F). Posttranslational modification in the form of protein ubiquitination is a dynamic and multifaceted process. When a ubiquitin molecule is attached to a substrate protein through the lysine site of the substrate, there are 7 lysine sites (commonly K6, K11, K27, K29, K33, K48, and K63) and 1 methionine site on the ubiquitin molecule that can be further linked to other ubiquitin molecules. We constructed plasmids containing single-site mutations in ubiquitin to investigate which polyubiquitin chain type is involved in PFKFB3 overexpression-mediated OPA1 ubiquitination. The data demonstrated that PFKFB3 overexpression mediated OPA1 ubiquitination, with only K6R (Lys6-Arg) Ubiquitin completely inhibiting PFKFB3-mediated OPA1 polyubiquitination (Fig. 7G). To further corroborate this finding, we employed K6-only Ubiquitin plasmids (which retains the K6 lysine residue while mutating the other six lysine residues to arginine), K11-only Ubiquitin plasmids, K27-only Ubiquitin plasmids, K29-only Ubiquitin plasmids, K33-only Ubiquitin plasmids, K48-only Ubiquitin plasmids and K63-only Ubiquitin plasmids. HEK293T cells were co-transfected with these mutated ubiquitin plasmids along with HA-OPA1 plasmids, with or without Flag-PFKFB3 plasmids. As shown in Fig. 7H, PFKFB3 predominantly augmented ubiquitination of the K6 type on OPA1. Moreover, we applied wild-type, K6, K6R, and K0 ubiquitin plasmids to verify the type of PFKFB3-mediated ubiquitination (Fig. 7I). Consistent with the previous results, K6-Ubiquitin promoted chain formation on OPA1, similar to wild-type ubiquitin (Fig. 7I). These results revealed that PFKFB3 overexpression stabilized OPA1 protein by promoting its K6-linked polyubiquitination.

The process of ubiquitination involves three enzyme types: ubiquitin-activating enzyme E1, ubiquitin-conjugating enzyme E2, and ubiquitin-protein ligase E3, in which an E3 ubiquitin ligase is substrate-specific. In addition, deubiquitinases are responsible for substrate protein deubiquitination. In this study, we utilized the Ubibrowser website to predict the E3 ubiquitin ligase and deubiquitinase of the OPA1 protein. We compared the predicted top 20 E3 ubiquitin ligase and the only deubiquitinase (CYLD) with the results obtained from IP-MS analysis of OPA1, respectively. Two E3 ubiquitin ligases were filtered out: NEDD4L and smurf1 (Fig. 7J–L). The results of the Co-IP experiment further confirmed the interaction between NEDD4L and OPA1 but not smurf1 (Fig. 7N). HEK293T cells were transfected with HA-OPA1 and cmyc-NEDD4L, with or without Flag-PFKFB3. The Co-IP assays revealed that PFKFB3 overexpression enhanced the interaction between OPA1 and NEDD4L (Fig. 7M). Next, we knocked down NEDD4L using small interference RNA, the sequence of si-2 was selected for further research (Fig. 7O, P). The knockdown of NEDD4L effectively inhibited the PFKFB3-mediated increase in OPA1 expression (Fig. 7Q, R). These results suggest that E3 ligase NEDD4L mediates the ubiquitination of OPA1 facilitated by PFKFB3 overexpression.

## Discussion

Diabetic cardiomyopathy (DCM) is a prominent cause of death in diabetics [34]. Despite advances in treatment, the prognosis of DCM remains unfavorable. In this study, we explored the distinct role of PFKFB3 overexpression in DCM and revealed several crucial findings: (1) PFKFB3 expression was reduced in the hearts of db/db mice. (2) Cardiac-specific overexpression of PFKFB3 significantly alleviated diabetic cardiac dysfunction and myocardial remodeling, associated with enhanced mitochondrial function and reduced cardiomyocyte apoptosis. (3) The knockdown of OPA1 attenuated the protective effects of PFKFB3 overexpression on diabetic cardiac dysfunction and myocardial remodeling in db/db mice. (4) Mechanistically, PFKFB3 stabilizes OPA1 expression by promoting its K6-linked polyubiquitination mediated by the E3 ligase NEDD4L, thereby preventing the degradation of OPA1 by the ubiquitin–proteasome system. Taken together, cardiac-specific overexpression of PFKFB3 significantly protected against DCM by enhancing myocardial OPA1 stability through NEDD4L-mediated atypical K6-linked polyubiquitination. Our study highlights PFKFB3/OPA1 as promising therapeutic targets for DCM.

The mitochondria serves as the powerhouse for all eukaryotic cells. Maintaining a dynamic balance between mitochondrial fission and fusion is crucial for preserving mitochondrial function. Diabetic hearts exhibit excessive mitochondrial fission and mitochondrial dysfunction characterized by reduced MMP, decreased ATP levels, and impaired OXPHOS, leading to excessive ROS production and apoptosis [35, 36]. Hence, mitochondrial dysfunction is considered a hallmark of DCM [37]. Chen et al. reported that IL-22 mitigated kidney damage by AMPK/AKT signaling and inhibiting mitochondrial dysfunction via PFKFB3 [16]. This study suggests a potential relationship between PFKFB3 and mitochondrial function, but the underlying mechanism remains unclear. In our study, PFKFB3 overexpression inhibited excessive mitochondrial fission and improved mitochondrial function. Mechanistically, PFKFB3 interacts with OPA1 and prevents its degradation in DCM. Hence, targeting PFKFB3/OPA1-mediated enhanced mitochondrial function represents a promising strategy for treating DCM.

The OPA1 gene, located on 3q28, spans over 100 kb and comprises 31 exons [38]. The OPA1 protein is involved in various functions, including promoting mitochondrial fusion, maintaining the respiratory chain [39] and membrane potential [40], organization of cristae, and regulating apoptosis [32]. Previous studies have emphasized the significance of OPA1 in DCM. Liu et al. revealed that paeonol induces OPA1-mediated mitochondrial fusion through the activation of the CK2α-Stat3 pathway in DCM [36]. Ding et al. reported that the mitochondrial fusion promoter-M1 restores mitochondrial dynamics balance and ameliorates DCM, which is dependent on OPA1 [33]. Furthermore, Punicalagin prevents DCM by promoting OPA1-mediated mitochondrial fusion via the PTP1B-Stat3 pathway [36, 41]. To further underscore the beneficial role of OPA1 in diabetic cardiomyopathy, we investigated the effects of OPA1 upregulation on apoptosis and ROS in AC16 cells. AC16 cells were transfected with either pcDNA3.1 or pcDNA3.1-HA-OPA1 plasmids, followed by PA or solvent control treatment. As shown in Fig. S9A–S9F, OPA1 overexpression significantly reduced apoptosis and the ROS production in PA-treated AC16 cells. These studies and our data demonstrate the close association between OPA1 and DCM, underscoring the potential of OPA1 as a promising therapeutic target for DCM.

Ubiquitination, the covalent attachment of ubiquitin to specific substrate proteins, is a multifunctional post-translational modification that affects various disease processes, including cardiovascular disease. Ubiquitination can take various forms, including monoubiquitination, multi-monoubiquitination, and polyubiquitination at specific lysine residues (K6, K11, K27, K29, K33, K48, and K63), as well as at the N-terminal methionine of ubiquitin. K48-linked chains are the predominant and extensively studied type of linkage involved in the degradation of ubiquitinated substrates by the UPS. In this process, substrates tagged with K48-linked chains are recognized, bound, and subsequently degraded by the 26S proteasome. K63-linked ubiquitination is the second most well-known and abundant type, implicated in endocytosis and the innate immune response. Other atypical ubiquitin sites, such as K11-linked ubiquitination, are associated with regulating the cell cycle or earmarking substrates for degradation. However, this type modulates protein–protein interactions or stabilizes proteins such as β-Catenin [42]. The DNA damage response has been repeatedly shown to be controlled by K6-linked ubiquitination [43, 44]. K6 and K63-linked chains play a crucial role in the autophagic processing of damaged mitochondria [45]. K27-linked ubiquitination plays a role in antimicrobial, antiviral immunity, and antifungal signaling [46]. The joint presence of K27 and K29-linked chains has been reported to promote proteasomal and autophagic degradation and protein aggregation associated with Parkinson’s disease [47, 48]. In our study, PFKFB3 overexpression stabilized OPA1 by promoting its K6-linked polyubiquitination mediated by the E3 ligase NEDD4L, thereby suppressing mitochondrial fragmentation and restoring mitochondrial function in db/db mice.

The mechanism by which PFKFB3 overexpression reduces ROS production in DCM may be multifaceted. On one hand, PFKFB3 mitigates oxidative stress in cardiac tissues of diabetic mice by interacting with OPA1 and inhibiting the downregulation of OPA1 expression, which leads to a reduction in oxidative stress. Chen et al. reported that the decrease in OPA1 expression resulted in increased levels of ROS [49]. We found that PFKFB3 could stabilize OPA1 expression, thereby reducing ROS production. On the other hand, PFKFB3 overexpression may alleviate oxidative stress by stimulating glycolysis. Previous studies have indicated that promoting glycolysis resulted in reduced oxygen consumption and ROS production [50]. In this study, when glycolysis was inhibited by 2-DG, the production of ROS increased (Fig. S5G). Hence, PFKFB3 may mitigate the generation of ROS by modulating both OPA1 and glycolytic pathways.

It is important to acknowledge that our study has certain limitations. Firstly, OPA1 predominantly exists at the mitochondrial membrane, while PFKFB3 is situated in the cytoplasm and nucleus, prompting a compelling question: How do they interact? Previous studies have reported that OPA1 is encoded by a nuclear gene, implying that the translational process of OPA1 occurs in the cytoplasmic ribosome [51]. Additionally, Kar et al. discovered that newly synthesized forms of OPA1 undergo ubiquitination [52]. In our study, PFKFB3 mediated the ubiquitination of OPA1. Consequently, we hypothesize that upon OPA1 synthesis in the cytoplasm, PFKFB3 interacts with OPA1 and recruits the E3 ubiquitin ligase NEDD4L, which mediates ubiquitination of OPA1, thereby escorting OPA1 from the cytosol to the mitochondria and protecting it from degradation by the proteasome. Nevertheless, the precise mechanisms require further clarification. Secondly, the db/db mice and AC16 cells used in our study may not entirely replicate the pathophysiology of patients with DCM. Thirdly, additional investigation is required to ascertain whether the protective benefits of PFKFB3 can be extended to other diabetic models, including female mice. Despite these limitations, our study provides valuable insights into the role of PFKFB3 in mitigating DCM.

## Conclusion

Cardiac-specific overexpression of PFKFB3 protects against DCM by enhancing myocardial OPA1 stability via NEDD4L-mediated atypical K6-linked polyubiquitination. Our findings imply that PFKFB3/OPA1 might be therapeutic targets for DCM.

## Supplementary information

Extended information about the Materials and Methods and Supplementary Figures can be accessed in the Supplementary Materials.

### Supplementary Information

Below is the link to the electronic supplementary material.

Supplementary file1 (DOCX 3909 KB)

## Data Availability

The datasets generated during and/or analysed during the current study are available from the corresponding author on reasonable request.

## Abbreviations

ATP: Adenosine triphosphate
AAV9: Adeno-associated virus serotype 9
Co-IP: Co-immunoprecipitation
CQ: Chloroquine
CHX: Cycloheximide
DCM: Diabetic cardiomyopathy
MMP: Mitochondrial membrane potential
MS: Mass spectrometry
OCR: Oxygen consumption rate
OXPHOS: Oxidative phosphorylation
OPA1: Optic atrophy 1
ECAR: Extracellular acidification rate
PFKFB3: 6-Phosphofructo-2-kinase/fructose-2,6-biphosphatase 3
HF: Heart failure
ROS: Reactive oxygen species
TUNEL: Terminal-deoxynucleotidyl transferase-mediated nick end labeling
DHE: Dihydroethidium
WGA: Wheat Germ Agglutinin
TEM: Transmission electron microscopy
