# Supplementary material for: Cardiac-specific PFKFB3 overexpression prevents diabetic cardiomyopathy via enhancing OPA1 stabilization mediated by K6-linked ubiquitination
Source: Cell Mol Life Sci. 2024 May 22;81(1):228. doi: 10.1007/s00018-024-05257-5 (PMC11111656; doi:10.1007/s00018-024-05257-5)
Supplement: Supplementary file 1 — Supplementary file1 (DOCX 3909 KB) [file 18_2024_5257_MOESM1_ESM.docx]

**Cardiac-specific PFKFB3 Overexpression Prevents Diabetic Cardiomyopathy via Enhancing OPA1 Stabilization Mediated by K6-linked Ubiquitination**

Jinlan Luo^1,2^, Shuiqing Hu^2^, Jingrui Liu^2^, Lili Shi^1^, Liman Luo^1^, Wenhua Li^1^, Yueting Cai^2^, Jiaxin Tang^1^, Siyang Liu^1^, Menglu Fu^1^, Ruolan Dong^3^, Yan Yang^4^, Ling Tu^1,5^* and Xizhen Xu^2,5^*

^1^Department of Geriatric Medicine, Tongji Hospital, Tongji Medical College, Huazhong University of Science and Technology, Wuhan 430030, China

^2^Division of Cardiology and Department of Internal Medicine, Tongji Hospital, Tongji Medical College, Huazhong University of Science and Technology, Wuhan 430030, China

^3^Institute of Integrated Traditional Chinese and Western Medicine, Tongji Hospital, Tongji Medical College, Huazhong University of Science and Technology, Wuhan 430030, China

^4^Health Management Center, Tongji Hospital, Tongji Medical College, Huazhong University of Science and Technology, Wuhan 430030, China

^5^Hubei Key Laboratory of Genetics and Molecular Mechanisms of Cardiological Disorders, Wuhan 430030, China

Corresponding authors:

To whom correspondence should be addressed to Dr. Xizhen Xu, MD, Ph.D. or Dr. Ling Tu, MD, Ph.D., Division of Cardiology, Department of Internal Medicine, Tongji Hospital, Tongji Medical College, Huazhong University of Science and Technology; Fax and Tel.: 86-27-83662843; E-Mail address: [xzxu@tjh.tjmu.edu.cn](mailto:xzxu@tjh.tjmu.edu.cn) or [lingtu@tjh.tjmu.edu.cn](mailto:lingtu@tjh.tjmu.edu.cn)

**Materials and methods**

**Animals**

8-week-old male leptin receptor-deficient (C57BLKS/J-LepRdb/LepRdb, db/db) mice and their control littermates (C57BLKS/J-LepRdb/+, db/m) were obtained from GemPharmatech (Nanjing, China). Approval for all animal care and experimental procedures was granted by the Experimental Animal Research Committee of Tongji Medical College, Huazhong University of Science and Technology (IACUC Issue No.: TJH-202109019). Our studies were conducted in strict adherence to the Guidelines from Directive 2010/63 EU of the European Parliament and ARRIVE guidelines. Before the experiment, there were no significant differences in body weight, fasting blood glucose, or food intake among the mice in different groups.

**Experimental design 1 (n=10/group)**

To investigate the potential beneficial effects of PFKFB3 in DCM, mice were randomly assigned to four groups:

(1) db/m+rAAV9-cTnT-GFP.

(2) db/m+rAAV9-cTnT-Flag-PFKFB3.

(3) db/db+rAAV9-cTnT-GFP.

(4) db/db+rAAV9-cTnT-Flag-PFKFB3.

**Experimental design 2 (n=10/group)**

To determine if PFKFB3 overexpression alleviated DCM by regulating OPA1, the mice were divided into five groups:

(1) db/m+rAAV9-cTnT-Vector.

(2) db/db+rAAV9-cTnT-Vector.

(3) db/db+rAAV9-cTnT-Flag-PFKFB3.

(4) db/db+rAAV9-cTnT-shOPA1.

(5) db/db+rAAV9-cTnT-Flag-PFKFB3+rAAV9-cTnT-shOPA1.

Recombinant Adeno-associated virus (rAAV) with the cardiomyocyte-specific promoter (cTnT) was constructed by DesignGene Biotechnology (Shanghai, China) and administered via mice tail vein in a volume of 150 μL for each mouse (5x10^12 vg/ml). Echocardiographic and hemodynamic evaluations were carried out at 20 weeks of age. Mice were anesthetized with isoflurane (2%, inhalation with a mask) and pentobarbital sodium (50 mg/kg, intraperitoneal injection) respectively. At the end of the experiment, pentobarbital sodium was administered via intraperitoneal injection, with 200 mg/kg for mice euthanasia. The heart and blood samples were collected for further research.

**Cell culture**

AC16 human cardiomyocyte cell line was obtained from Procell (Procell Life Science & Technology Co., Ltd) and HEK-293T cells were obtained from ATCC (Manassas, VA, Designation No. PTA-1500). The cells were cultured in DMEM medium, supplemented with 10% fetal bovine serum (FBS, Gibco). The adenovirus-PFKFB3 and adenovirus-GFP were obtained from Hanbio (Shanghai, China). The overexpression plasmid, siRNA and corresponding negative control were ordered from DesignGene Biotechnology (Shanghai, China). The transfection reagent used for the transient transfer was Lipofectamine 2000 (Invitrogen, Carlsbad, CA) according to the manufacturer's recommendations. After transfection, AC16 cells were treated with 200 μM PA for 48 hours.

**Echocardiography and hemodynamics**

Transthoracic echocardiography was performed noninvasively using a vevo 1100 high-resolution imaging system (VisualSonics, Toronto, ON, Canada). Briefly, mice were lightly anaesthetized with 2% isoflurane and set in a supine position on a heated physiological information monitoring platform (37℃). The chest hairs were shaved, and a layer of acoustic coupling gel was applied to the thorax. B-mode echocardiograms from the parasternal long-axis view were acquired to obtain the maximum left ventricular (LV) length. Then, rotate the ultrasound probe 90 degrees clockwise to capture parasternal short-axis images at the papillary level. An M-mode recording of the LV was collected using two-dimensional (2D) echocardiographic guidance in both the short and long-axis views. The following structural variables were assessed: averaged LV systolic and diastolic anterior wall thickness (LVAWs, LVAWd), LV diastolic and systolic posterior wall thickness (LVPWd, LVPWs), interventricular septal end-diastole and end-systole (IVSd, IVSs) and LV diastolic and systolic internal dimensions (LVIDd, LVIDs) etc. LV ejection fraction (EF) and LV fractional shortening (FS) were calculated as follows: EF= (LV Vol; d-V Vol; s)/LV Vol; d×100%, FS= (LVIDd – LVIDs)/LVIDd×100%. To evaluate LV diastolic function, adjust the platform and ultrasonic probe. The four-chamber section of the heart was used to find the aortic blood flow in color Doppler mode, and the early diastolic blood flow filling (peak E) and the peak atrial systolic blood flow (peak A) were observed and recorded in pulsed Doppler mode. Each of these captured image loops included about 20 cardiac cycles, and all parameters were averaged over 3 cardiac cycles per loop for analyses. At the end of the echocardiography evaluation, a sterile 1.0-Fr Millar Mikro-Tip catheter transducer (Millar 1.4F, SPR 835, Millar Instruments, Houston, TX) was inserted into LV through the right carotid artery. Hemodynamic parameters, including LV systolic pressure (LVSP), LV end-diastolic pressure (LVEDP), and the maximal and minimal first derivative of LV pressure, were recorded and analyzed with PowerLab and LabChart software. All studies and analyses were performed blinded to different treatments.

**Transmission electron microscopy (TEM)**

Heart samples from the left ventricle were isolated (≤1 mm) and immersed in 2.5% glutaraldehyde phosphate buffer for 2 hours at room temperature, rinsed with 0.1 M phosphate buffer for 3 times, post-fixed with 1% osmium acid buffer for 2 hours. After dehydration with different acetone concentrations, embedding, ultrathin sections (60 nm) were prepared. The slices were stained with uranium acetate and lead citrate and observed by electron microscopy (HT7800, HITACHI, Japan). Mitochondrial images were analyzed using Image J software (National Institutes of Health, USA).

**Assessment of mitochondrial morphology in cardiomyocytes**

The mitochondria in the cardiomyocytes were labeled with 25 nmol/L MitoTracker Red CMXRos probe (M7512, Invitrogen by Thermo Fisher Scientific, Carlsbad, USA) and captured by ZEN 2.1 software (Carl Zeiss; Oberkochen, Germany). The images were quantified via the Image J software (National Institutes of Health, USA).

**Biochemical assays**

Fasting blood glucose levels were measured with a One-Touch VerioVue glucometer (Lifescan, Johnson & Johnson, Shanghai, China). Blood samples were obtained from the mouse tail vein. The serum lipids: serum total cholesterol (TC), triacylglycerol (TG), high-density lipoprotein cholesterol (HDL-C), and low-density lipoprotein cholesterol (LDL-C) were measured using Roche Cobas 8000 automatic biochemical analyzer (Roche Diagnostics GmbH, Mannheim, Germany). Intraperitoneal glucose tolerance tests (GTT) and Insulin resistance tests (ITT) were performed as described previously (1). Lactic acid levels of cardiac tissue were detected using assay kits from Nanjing Jiancheng Bioengineering Institute according to the manufacturer's protocols.

**RNA extraction and Real-time quantitative PCR**

Total RNA was extracted from frozen cardiac tissues using TRIzol Reagent ((Invitrogen, 15596026CN, Carlsbad, CA, USA) according to the manufacturer's instructions. Complimentary DNA (cDNA) was obtained by reverse transcription of 1.0 mg RNA via a HiScript® III RT SuperMix kit (Vazyme Biotech Co., Ltd., R211-02, Nanjing, China). Quantitative real-time PCR analyses were performed with the ChamQ SYBR qPCR master mix reagents (Vazyme Biotech Co., Ltd., Q331-02, Nanjing, China) on a 7900HT FAST Real-Time PCR System (Life Technologies, Carlsbad, CA). The target mRNA levels were determined using the 2-ΔΔCT method and normalized to the housekeeping gene β-actin. The primer sequences for rt-glycoqPCR are as follows.

| Genes | Forward primer (5'-3') | Reverse primer (5'-3') |
| --- | --- | --- |
| PFKFB3 | ACCAAAGATCACCCACGGATGT | TGACCTTGGTGACCTCTTCTTG |
| OPA1 | GGTGACTCTGAGTGAAGGTCCT | TGGTCTCAGGGCTAACGGTA |
| β-actin | TGACGTGGACATCCGCAAAG | CTGGAAGGTGGACAGCGAGG |

Supplementary table 1

**Western blot assay**

Cardiac tissues and cultured cells were lysed with RIPA Lysis Buffer (MedChemExpress, HYK1001, Monmouth Junction, NJ, USA) containing general protease and phosphatase inhibitors. Western blot was performed utilizing the standard method as described previously (2). The antibodies and dilution ratios used in this study are listed in Supplementary Table 2. Protein bands were visualized using the enhanced chemiluminescence detection reagent. Finally, the ImageJ program (National Institutes of Health software, USA) was used to determine the band density. The target protein abundance was normalized to respective β-actin levels.

**Co-immunoprecipitation (Co-IP)**

Co-IP was used to evaluate whether two proteins interacted. In brief, AC16 cells or HEK293T cells were lysed with RIPA lysis buffer containing 50 mM Tris (pH 7.4), 150 mM NaCl, 1% Triton X-100, 1% sodium deoxycholate, 0.1% SDS, and a mixture of protease and phosphatase inhibitors. The cell lysate was centrifuged at 12,000g at 4°C for 15 min, and the supernatant was collected. Note that 1/4 volume of supernatant was taken out as input. Following a 2-hour incubation at 4°C on a rotator with the primary antibody or IgG, the protein A/G magnetic beads should be washed three times. Then the prepared supernatant was added and bound with the beads-antibody complex overnight at 4°C with gentle rotation. Finally, the beads were collected and washed for 5 times. The target proteins were eluted through boiling in 1 × loading buffer at 95°C for 10 min followed by immunoblotting analyses, companying with Coomassie Blue Super Staining (Beyotime, P0017F, Shanghai, China) or mass spectrometry analyses.

**Mass spectrometry (MS)**

MS was performed by SpecAlly Life Technology Co., Ltd. (Wuhan, China). In brief, the beads samples obtained from the immunoprecipitation experiment were washed and incubated in the reaction buffer (1% SDC/100 mM Tris-HCl, pH 8.5/10 mM TCEP/40 mM CAA) at 95°C for 10 min for protein denaturation, cysteine reduction, and alkylation. Then the protein was digested, purified, and vacuum-dried. LC-MS/MS data acquisition was carried out on a Q Exactive Plus mass spectrometer coupled with an EASY-nLC 1200 system (both Thermo Scientific). The original data were analyzed with MaxQuant (V1.6.6) software using the Andromeda database search algorithm. The target proteins were identified by searching against the UniProt (The Universal Protein Resource) database.

**Histological Analyses**

Myocardial tissue was freshly collected and fixed in 4% paraformaldehyde overnight. The samples were dehydrated, embedded in paraffin, and sectioned into 4 μm thick transverse sections. Collagen deposition was assessed using Masson (Solarbio Life Sciences, G1346, Beijing) and Sirius red (Solarbio Life Sciences, S8060, Beijing) staining according to the manufacturer's instructions. Wheat Germ Agglutinin (WGA) staining (Vector Labs, RL-1022-5, Newark, California, United States) was used to assess cardiomyocyte size. TUNEL staining was performed to evaluate cardiomyocyte apoptosis via TUNEL Detection Kit (KeyGEN BioTECH, KGA702-1, Nanjing, China). For immunohistochemistry, sections were deparaffinized, rehydrated, antigen recovered, and treated with 3% H_2_O_2_ for 30 min. After blocking, samples were incubated with primary antibody overnight at 4°C and followed with horseradish peroxidase (HRP)-conjugated secondary antibodies for 2 h at room temperature. A diaminobenzidine (DAB) solution was used to visualize the reaction. For DHE staining, myocardial tissue was embedded with cryo-embedding media (OCT), sectioned into 10-μm thickness using a cryotome and stained with dihydroethidium ((Beyotime, S0063, Shanghai, China) at 37°C for 30 min. Images were captured by ZEN 2.1 software (Carl Zeiss; Oberkochen, Germany) and quantified via the Image-Pro Plus software (version 6.0; Media Cybernetics, Bethesda, MD, USA).

**ATP Assays**

The ATP levels were determined via an Enhanced ATP Assay Kit (Beyotime, S0027, Shanghai, China) according to the manufacturer's instructions. Briefly, cells/tissue were lysed with ATP lysis buffer on ice for 10 min and centrifuged at 12,000 g at 4°C for 5 min. Add 100 µL of ATP Assay Working Solution to each well of the 96-well opaque plate (LABSELECT, 11514, Anhui, China) and place at room temperature for 3-5 min. Then transfer 20 µL of sample supernatant or standard to the assay well and mix quickly. A microplate reader ((BioTek Inc., Synergy2, Vermount, USA)) was applied to detect the luminescence (RLU) of each well. The ATP concentration was calculated based on its corresponding RLU from the standard curve. All ATP levels were normalized to the total protein of each sample.

**OCR and ECAR**

A Seahorse XFe24 Analyzer (Agilent, Les Ulis, France) was used to evaluate glycolytic flux and the function of mitochondria by measuring the extracellular acidification rate (ECAR) and the oxygen consumption rate (OCR). Briefly, AC16 cells were seeded in an Agilent Seahorse XF24 Cell Culture Microplate at a density of 5×10^4 cells per well except for four wells, which serve as "blank" wells. The sensor cartridge was hydrated overnight in a non-CO2 37°C incubator. Before the test, the culture medium was replaced with an assay medium supplemented with glucose (10 mM), sodium pyruvate (1 mM), and glutamine (2 mM). The OCR was measured following the sequential addition of oligomycin (1 μM), FCCP [Carbonyl cyanide 4-(trifluoromethoxy) phenylhydrazone (1.5 μM)], and rotenone + antimycin A (0.5 μM), using Cell Mito Stress Test Kit (Agilent, Santa Clara, CA). For ECAR measurement, the culture medium was replaced with an assay medium supplemented with fresh glutamine (2 mM) before the test. The measurement was performed with sequential injections of glucose (10 mM), oligomycin (1 µM), and 2-deoxyglucose (2-DG) (50 mM) using Seahorse XF Glycolysis Stress Test Kit (Agilent, Santa Clara, CA). All OCR and ECAR values were normalized to the total protein concentrations from the corresponding wells and analyzed with the software Wave 2.6.1 (Agilent, Les Ulis, France).

**Flow cytometry analyses**

The rate of apoptotic cells was identified via the Annexin V-FITC Apoptosis Detection kit (Beyotime, KGA108, Nanjing, China), the mitochondrial membrane potential (MMP) was detected using JC-1 Assay Kit (Beyotime, C2003S, Nanjing, China), the intracellular reactive oxygen species (ROS) production was assessed using Reactive Oxygen Species Assay Kit (Beyotime, S0033S, Nanjing, China) according to the manufacturer's instructions. Flow cytometric analyses were performed immediately after staining on a CytoFLEX flow cytometer (Beckman Coulter, Brea, CA). Data were analyzed using CytExpert software (Beckman Coulter, Brea, CA).

**Statistical analyses**

The data is expressed as mean ± SD and statistical analyses were carried out using an unpaired Student's t-test for comparisons between two groups. For multiple groups, we employed one-way ANOVA followed by Tukey post hoc analysis. All analyses were performed with GraphPad Prism version 8.0.2 (GraphPad Software, La Jolla, CA). Statistical significance was denoted by P < 0.05.

References

1. Luo J, Hu S, Fu M, Luo L, Li Y, Li W, Cai Y, Dong R, Yang Y, Tu L, Xu X. Inhibition of soluble epoxide hydrolase alleviates insulin resistance and hypertension via downregulation of SGLT2 in the mouse kidney. The Journal of biological chemistry 2021;**296**:100667

2. Luo L, Luo J, Cai Y, Fu M, Li W, Shi L, Liu J, Dong R, Xu X, Tu L, Yang Y. Inulin-type fructans change the gut microbiota and prevent the development of diabetic nephropathy. Pharmacological research 2022;**183**:106367

**Figure legends**

**Fig. S1**

**A.** Representative Western blot of PFKFB2 expression in hearts of 20-week-old db/db mice and db/m mice. **B.** Quantitative analysis of PFKFB2 expression in hearts of 20-week-old db/db mice and db/m mice. n=4/group. Data are expressed as mean ± standard deviation (SD). p < 0.05 was considered significant.

**Fig. S2**

**A.** Body weight (g) (n=9/group). **B.** Fasting blood glucose (mmol/L) after a 16-h fasting (n= 8/group). **C** and **D.** Intraperitoneal glucose tolerance test (GTT; 2 g/kg, n= 6/gruop) and the area under the curve of GTT. **E** and **F**. Insulin tolerance tests (ITT; 0.75 U/kg, n=6 /group) and the area under the curve of ITT. Data are shown as mean ± SD. p < 0.05 was significant. * means p < 0.05 between db/m+rAAV9-cTnT-GFP and db/db+rAAV9-cTnT-GFP.

**Fig. S3**

**A.** Flow cytometry analysis of apoptosis by Annexin V-FITC and propidium iodide (PI) staining and quantification of apoptotic cells in AC16 cells with treatment as indicated (**D**), n=4/group. **B.** Flow cytometry analysis of ROS by DCFH-DA staining and quantification analysis of ROS production (**E**), n=4/group. **C.** and **F.** Representative Western blot of apoptosis-related protein: Cleaved caspase-3, Bax and anti-apoptotic protein Bcl-2 and associated quantitation analysis, n=3/group**.** Data are expressed as mean ± SD. p < 0.05 was considered significant.

**Fig. S4**

**A.** Representative confocal microscope images of mitochondrial morphology stained by MitoTracker Red, Scale bars, 50 μm, 10 μm. **B.** Quantification analysis of mean mitochondrial volume. **C.** Flow cytometry analysis of mitochondrial membrane potential by JC-1(tetrachloro-tetraethyl benzimidazol carbocyanine iodide) staining in AC16 cells. A high level of green fluorescence (x-axis) represents reduced mitochondrial membrane potential (ΔΨm) and a high level of red fluorescence (y-axis) represents normal ΔΨm. The green fluorescence rate was analyzed (**D**). **E** and **F.** Oxygen consumption rate (OCR) and quantitative statistical analysis of OCR. n = 3-6/group. Data are expressed as mean ± SD. p < 0.05 was considered significant.

**Fig. S5**

**A.** Lactic acid levels in cardiac tissues. n=6/group. **B.** Extracellular acidification rate (ECAR) showing glycolytic function using an XF Extracellular Flux Analyzer. **C.** Quantification analysis of glycolytic function parameters, values are normalized to protein concentration (n=4/group). **D.** Flow cytometry analysis of apoptosis by Annexin V-FITC and propidium iodide (PI) staining and quantification of apoptotic cells (**F**). AC16 were pretreated with 2-DG, an inhibitor of glycolysis (4 mM/mL) for 3 hours and then treated as previously described. **E.** Flow cytometry analysis of ROS by DCFH-DA staining and quantification of ROS production (**G**), n=6/group. Data are expressed as mean ± SD. p < 0.05 was considered significant.

**Fig. S6**

**A** and **B.** Quantitative analysis of immunohistochemical staining of PFKFB3 and OPA1 in the hearts of 20-week-old mice with different recombinant adeno-associated virus treatments. IOD, integrated optical density. n=6/group. **C** and **D.** Representative Western blot and associated quantitative analysis of OPA1 expression in hearts of 20-week-old mice. **E** and **F.** Representative Western blot and associated quantitative analysis of PFKFB3 expression in hearts of 20-week-old mice. Data are expressed as mean ± SD. n=3/group. p < 0.05 was considered significant.

**Fig. S7**

**A.** and **D.** Flow cytometry analysis of Annexin V-FITC and propidium iodide (PI) staining in AC16 cells and associated quantification of apoptosis cells. **B.** and **C.** Flow cytometry analysis of ROS by DCFH-DA staining and quantification of ROS fluorescence. n=4/group. Data are expressed as mean ± SD. p < 0.05 was considered significant.

**Fig. S8**

**A.** Representative confocal microscope images of mitochondrial morphology stained by MitoTracker Red. Scale bars, 50 μm, 25 μm. **B.** Flow cytometry analysis of mitochondrial membrane potential by JC-1 staining. **C.** Quantification of mean mitochondrial volume, n=4/group. **D.** Quantification of mitochondrial membrane potential. A high level of green fluorescence (x-axis) represents reduced mitochondrial membrane potential (ΔΨm) and a high level of red fluorescence (y-axis) represents normal ΔΨm. The green fluorescence rate was analyzed. n=4/group. Data are expressed as mean ± SD. p < 0.05 was considered significant.

**Fig. S9**

**A.** Flow cytometry analysis of apoptosis by Annexin V-FITC and propidium iodide (PI) staining and quantification of apoptotic cell rates in AC16 cells (**E**). **B** and **D.** Representative Western blot of OPA1 and associated quantitation analysis, **C** and **F.** Flow cytometry analysis of ROS by DCFH-DA staining and quantification of ROS production. n=3-5/group**.** Data are expressed as mean ± SD. p < 0.05 was considered significant.

**Supplementary table 2**

The source and dilution ratios of antibodies used in this study.

| **Target antigen** | **Source** | **Catalog #** | **Working concentration** |
| --- | --- | --- | --- |
| PFKFB3 | Abcam | Ab181861 | 1:1000 IP 1μg |
| PFKFB2 | Abclonal | A9311 | 1:1000 |
| Cleaved caspase-3 | Abmart | T40044F | 1:500 |
| Bcl-2 | Abmart | T40056S | 1:1000 |
| Bax | Abmart | T40051F | 1:2000 |
| OPA1 | Abclonal | A9833 | 1:2000 IP 1μg |
| HA | Abmart | M20003M | 1:3000 IP 1μg |
| Flag | Abmart | M20008S | 1:3000 IP 1μg |
| myc | Abclonal | AE010 | 1:5000 |
| Ubiquitin | Abclonal | A19686 | 1:1000 |
| His | Abclonal | AE068 | 1:1000 |
| PGC1α | Abclonal | A11971 | 1:1000 |
| NDUFS1 | Abclonal | A16926 | 1:1000 |
| SDHA | Abcam | Ab14715 | 1:1000 |
| UQCRC2 | Abcam | ab14745 | 1:1000 |
| MTCO2 | Abclonal | A11522 | 1:1000 |
| ATP5A1 | Proteintech | 66037–1-lg | 1:2000 |
| NEDD4L | Abclonal | A9078 | 1:1000 |
| Smurf1 | Abclonal | A16559 | 1:1000 |
| HRP Goat Anti-Rabbit IgG (H+L) | Abclonal | AS014 | 1：10000 |
| HRP Goat Anti-Mouse IgG (H+L) | Abclonal | AS003 | 1:10000 |

**Supplementary table 2**


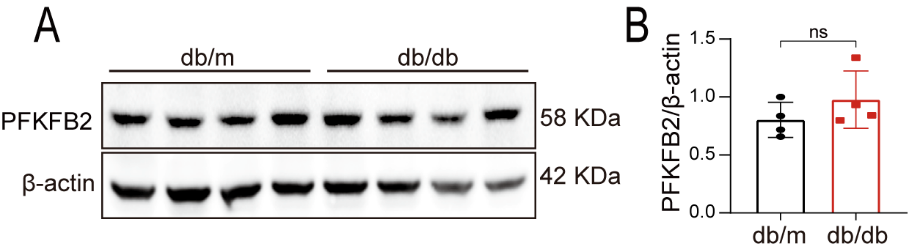


**Fig. S1**


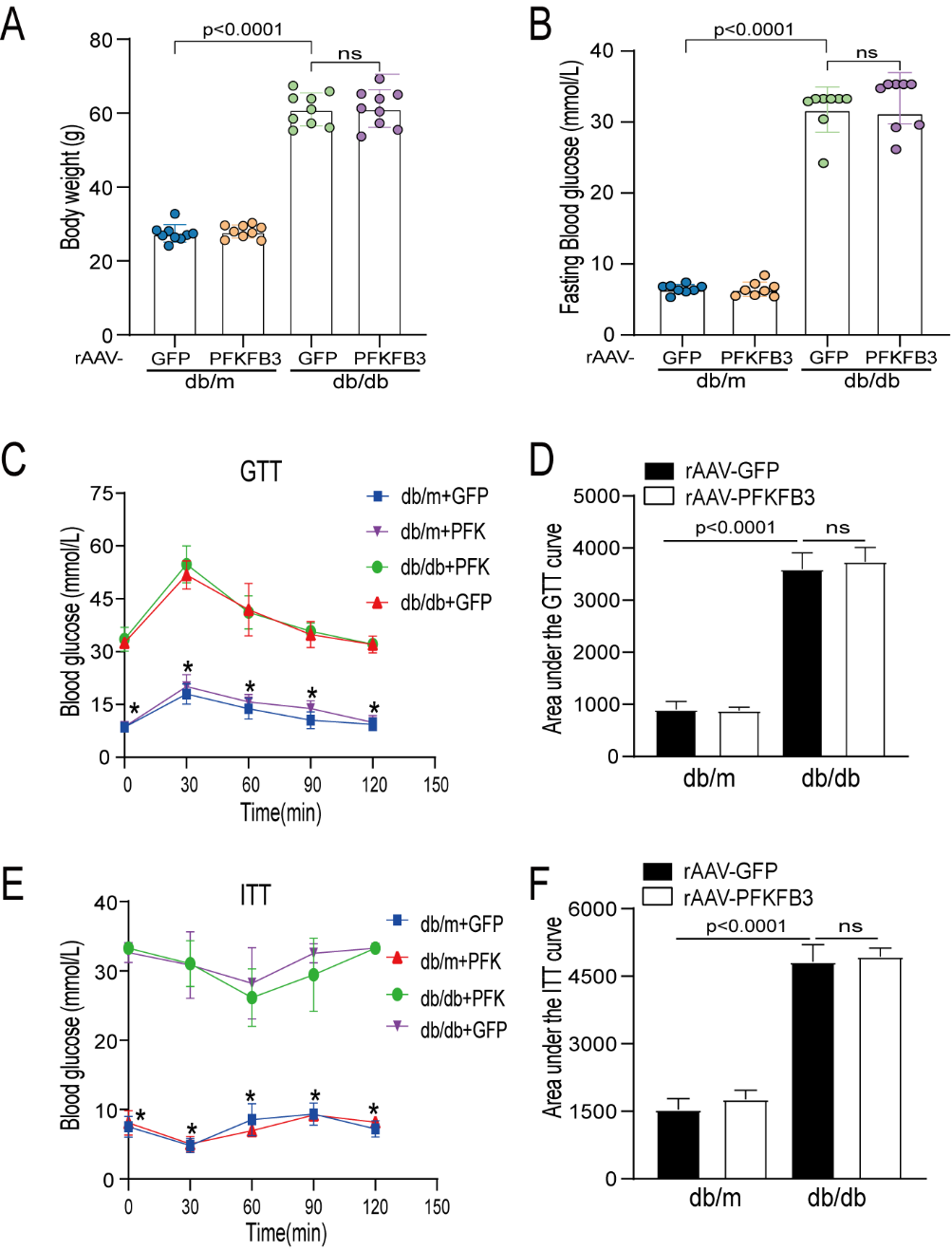


**Fig. S2**

**
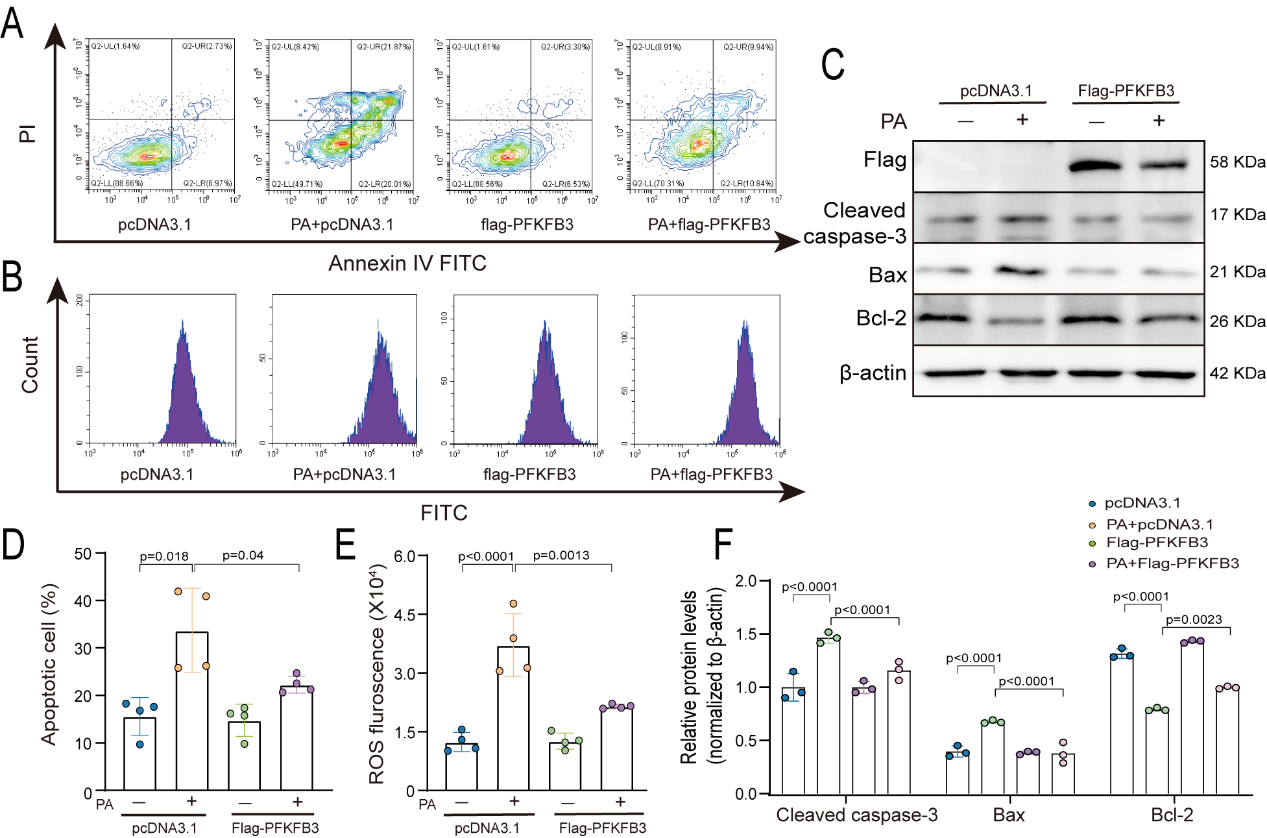
**

**Fig. S3**


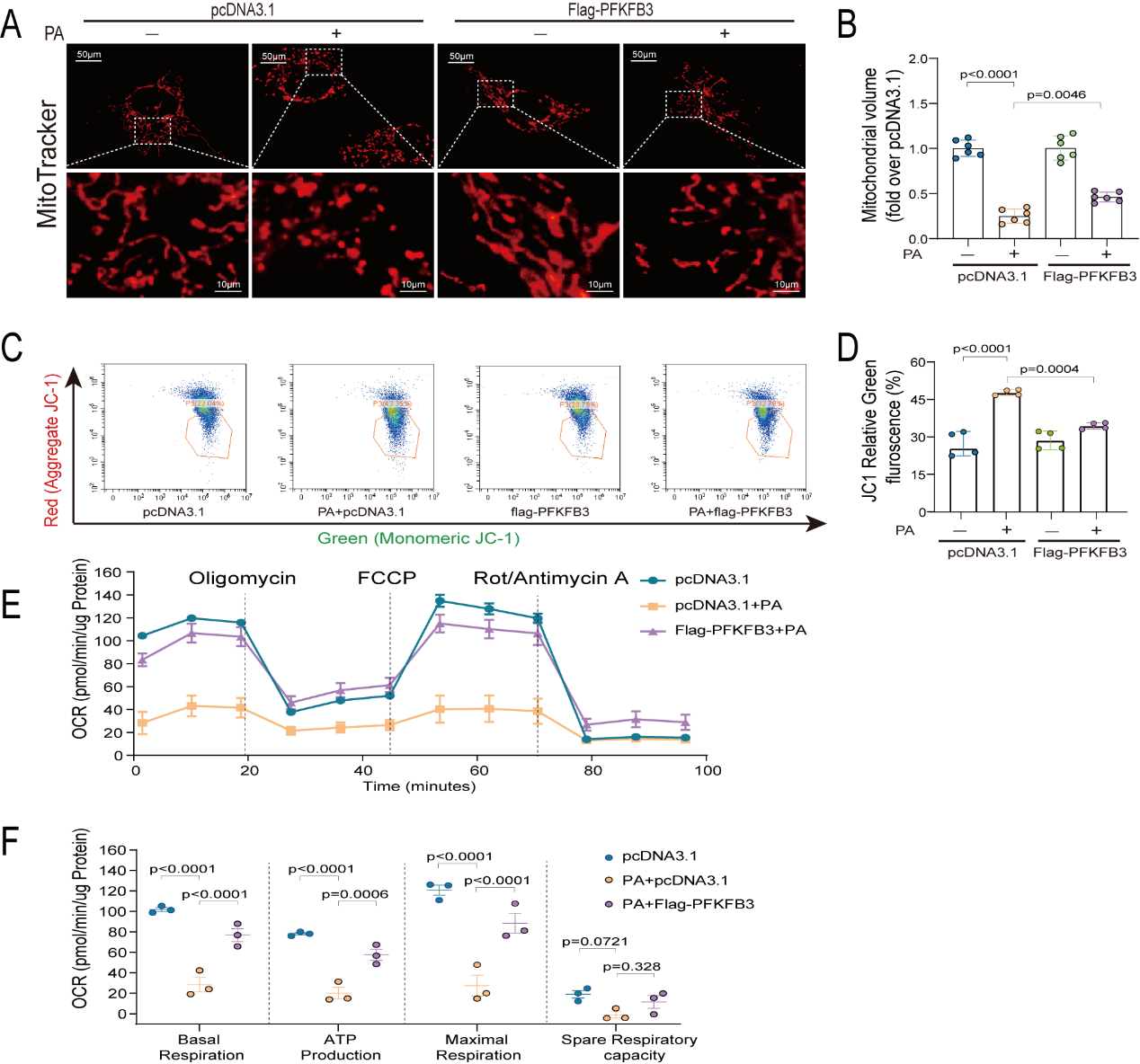


**Fig. S4**


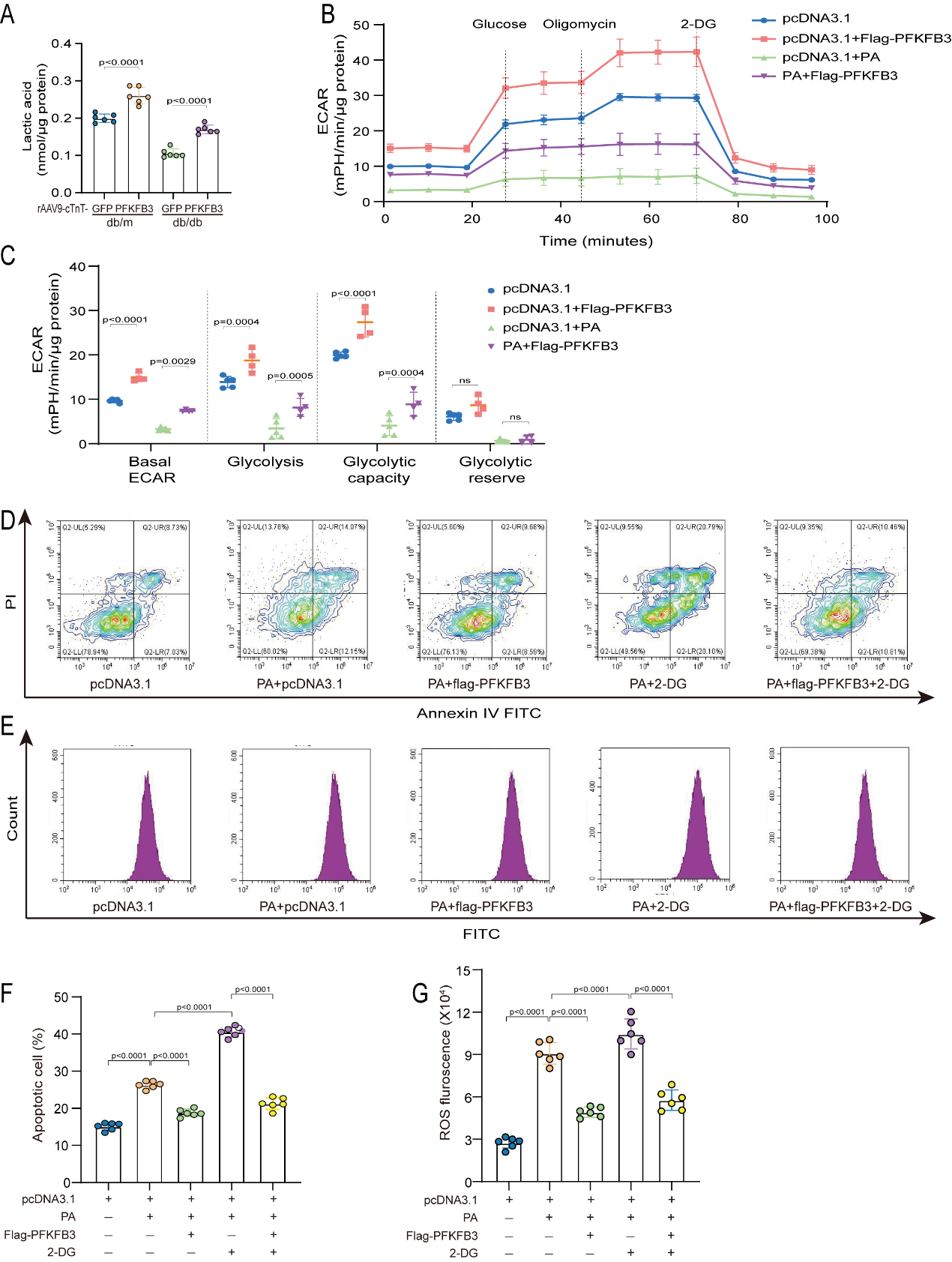


**Fig. S5**


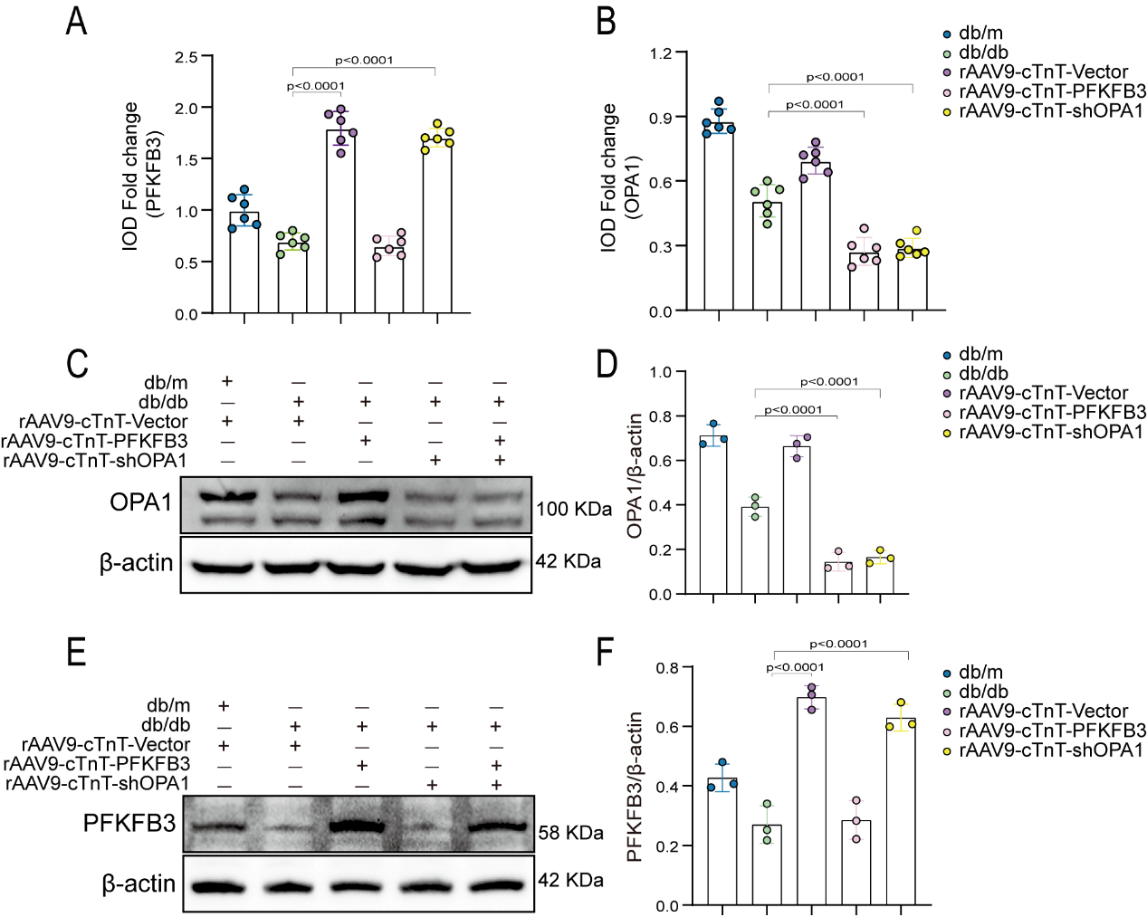


**Fig. S6**


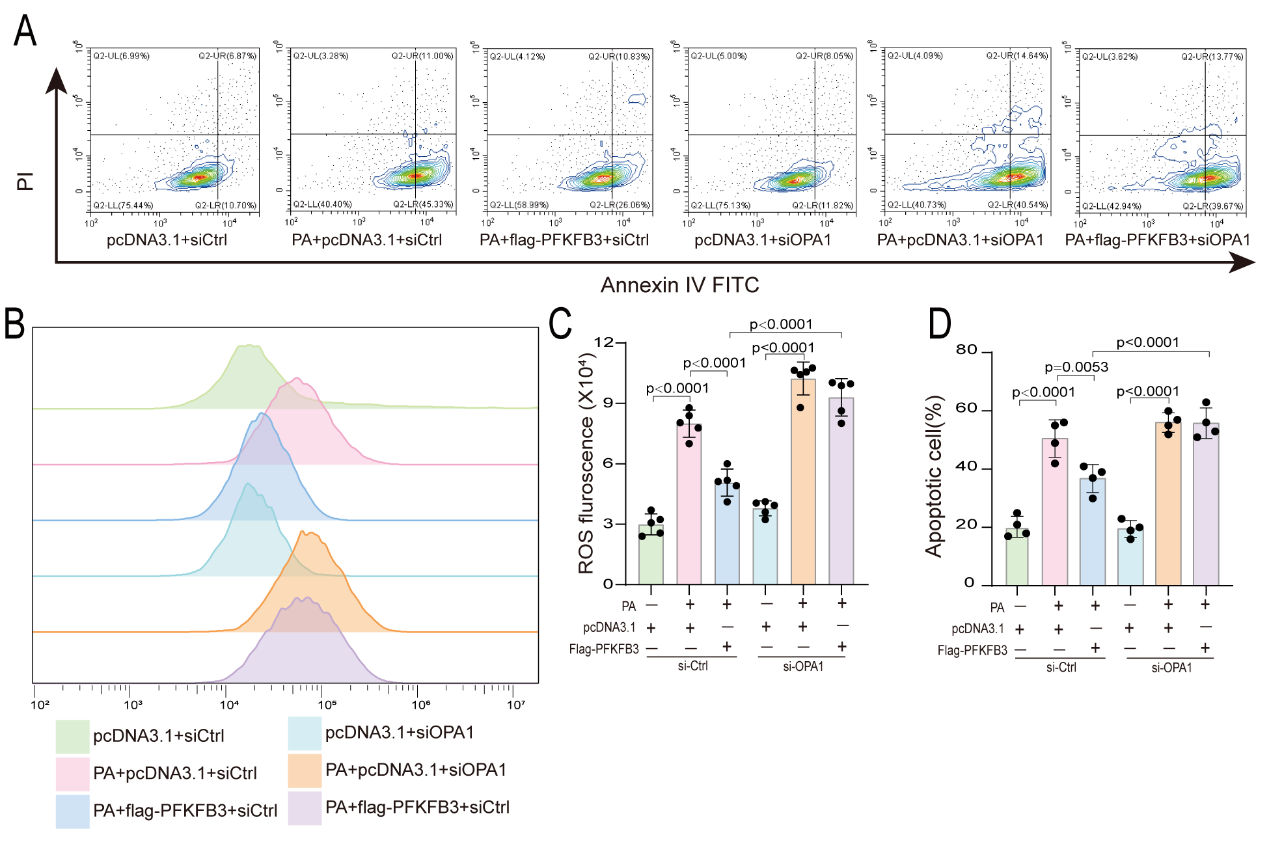


**Fig. S7**


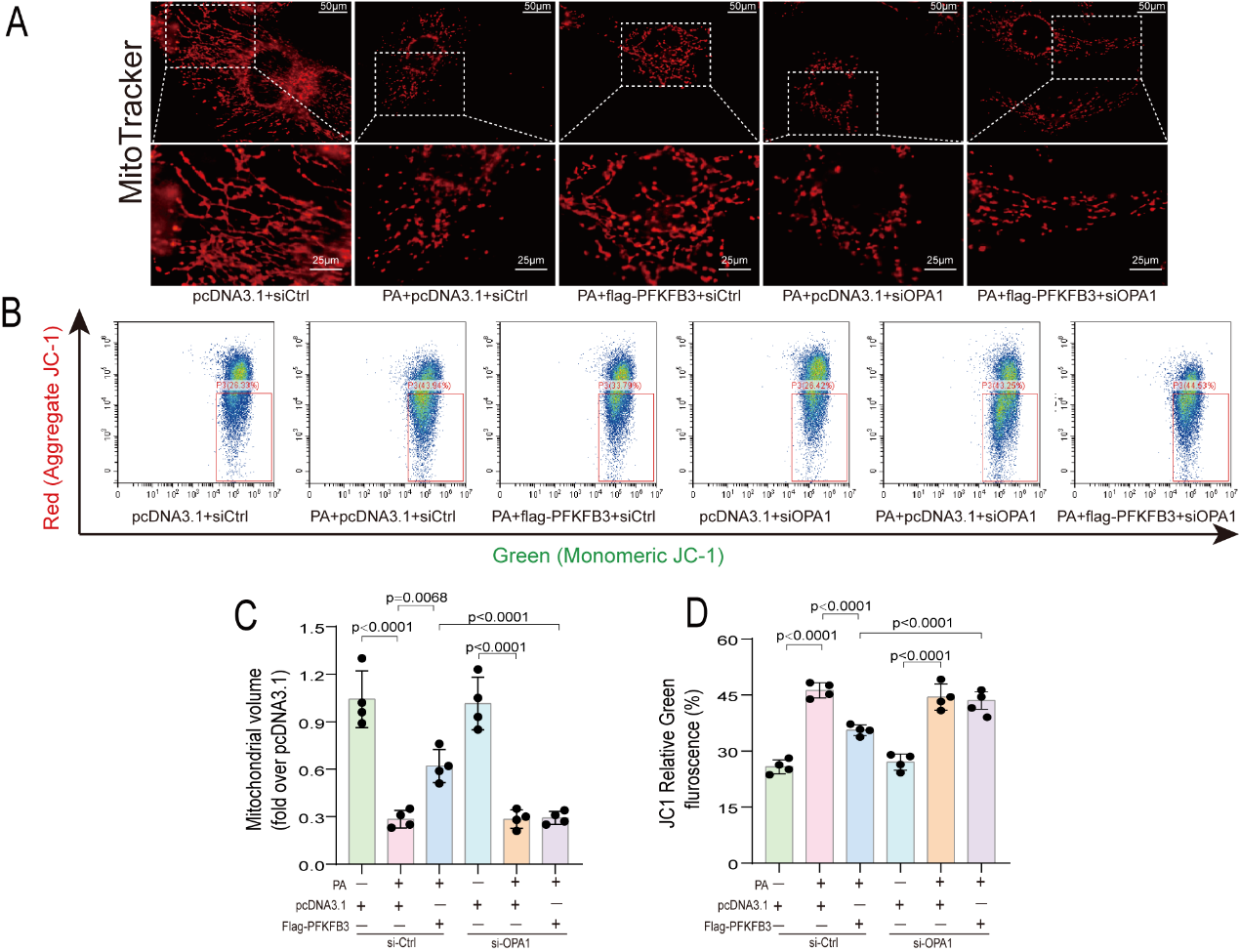


**Fig. S8**


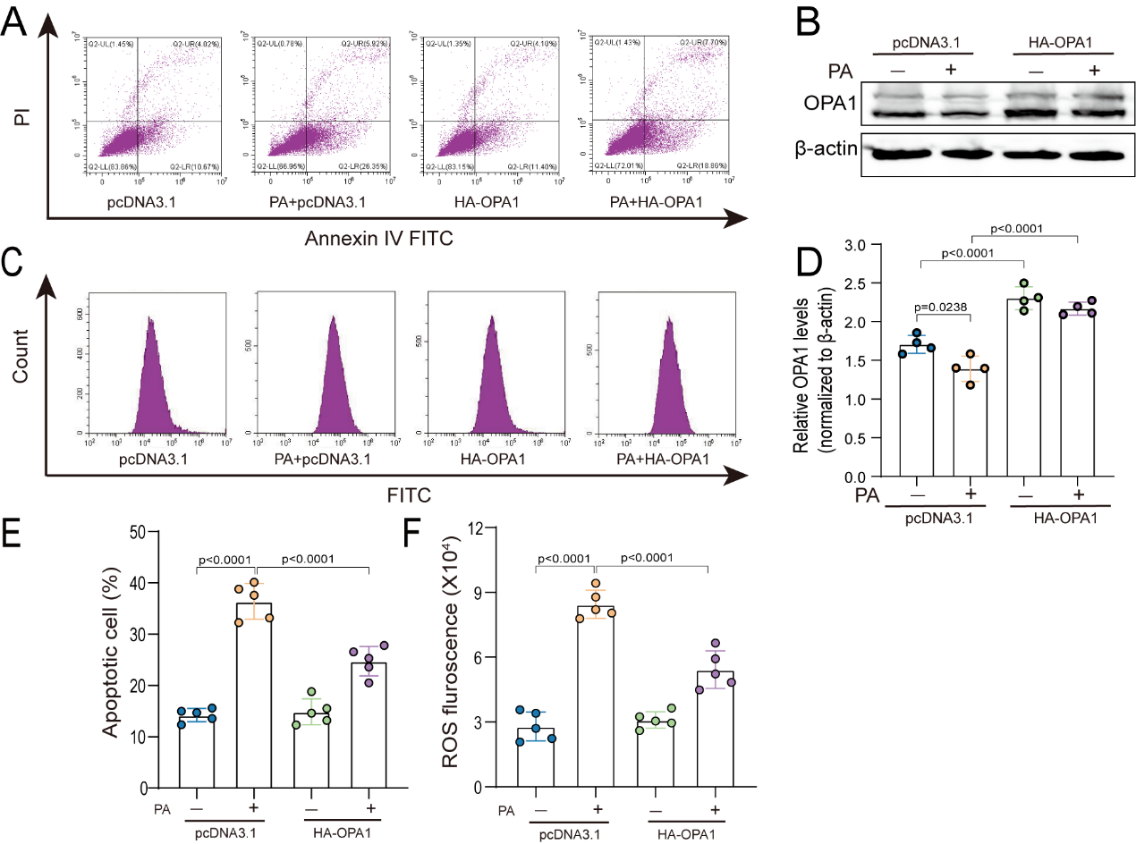


**Fig. S9**
